# A hub-and-spoke nuclear lamina architecture in trypanosomes

**DOI:** 10.1242/jcs.251264

**Published:** 2021-06-21

**Authors:** Norma E. Padilla-Mejia, Ludek Koreny, Jennifer Holden, Marie Vancová, Julius Lukeš, Martin Zoltner, Mark C. Field

**Affiliations:** 1 School of Life Sciences, University of Dundee, Dundee DD1 5EH, UK; 2 Institute of Parasitology, Biology Centre and Faculty of Sciences, University of South Bohemia, 37005 České Budějovice, Czech Republic; 3 Department of Parasitology, Faculty of Science, Charles University in Prague, BIOCEV 252 50, Vestec, Czech Republic

**Keywords:** Lamina, Macromolecular assembly, Trypanosomatid, Nuclear organization, Heterochromatin

## Abstract

The nuclear lamina supports many functions, including maintaining nuclear structure and gene expression control, and correct spatio-temporal assembly is vital to meet these activities. Recently, multiple lamina systems have been described that, despite independent evolutionary origins, share analogous functions. In trypanosomatids the two known lamina proteins, NUP-1 and NUP-2, have molecular masses of 450 and 170 kDa, respectively, which demands a distinct architecture from the ∼60 kDa lamin-based system of metazoa and other lineages. To uncover organizational principles for the trypanosome lamina we generated NUP-1 deletion mutants to identify domains and their arrangements responsible for oligomerization. We found that both the N- and C-termini act as interaction hubs, and that perturbation of these interactions impacts additional components of the lamina and nuclear envelope. Furthermore, the assembly of NUP-1 terminal domains suggests intrinsic organizational capacity. Remarkably, there is little impact on silencing of telomeric variant surface glycoprotein genes. We suggest that both terminal domains of NUP-1 have roles in assembling the trypanosome lamina and propose a novel architecture based on a hub-and-spoke configuration.

## Abstract (precis)

**Summary:** The arrangement of the lamina in trypanosomatids has architectural differences to the metazoan system.

## INTRODUCTION

The nucleus is delineated by a double lipid membrane bilayer, the nuclear envelope (NE), and is supported by a proteinaceous lamina that influences nuclear shape, size and resilience to physical forces together with mechano-signalling capability (Gruenbam and Foisner, 2015; Swift and Discher, 2014). The lamina also interacts with the nuclear pore complex (NPC), thereby influencing the position, function, organization and modification of chromatin (Aaronson et al., 1975; Goldberg and Allen, 1996; Liu et al., 2000). Moreover, the lamina governs epigenetic regulation, DNA replication, transcription and the cell cycle, and thus is a major organizing principle within the cell (Verstraeten et al., 2007; Zheng et al., 2018; Kim et al., 2019). Most lamina-dependent processes are important to all eukaryotic lineages, making the ability to build a lamina from distinct sets of proteins a remarkable example of convergent evolution (Koreny and Field, 2016). Moreover, many organisms lack any known lamina system, implying that yet more diversity remains to be uncovered.

In mammals, the lamina is comprised of ∼60 kDa lamin proteins of two major subtypes, lamin A and lamin B. B-type lamins are expressed in all mammalian nucleated cells (including germline and stem cells), whereas A-type lamins (which includes lamins A and C; splice variants that are both encoded by *LMNA*) have a restricted expression profile, and are restricted to differentiated cells (Lehner et al., 1987; Rober et al., 1989; Constantinescu et al., 2006). Lamins form homotypic filaments distributed throughout the nucleus with the separate networks interacting in a complex manner (Goldberg et al., 2008; Shimi et al., 2008, 2015; Turgay et al., 2017; Nmezi et al., 2019). However, lamin B is more intimately associated with the inner NE, whereas lamin A faces the nucleoplasm and avoids regions of the NE proximal to NPCs. B-type lamin filaments are thinner (7.3±0.9 nm; mean±s.d.) than A-type (16±1.7 nm) as determined by expression in *Xenopus* oocytes (Goldberg et al., 2008). Lamin B is highly ordered into layers and related to stabilization of nuclear shape, whereas lamin A forms bundles and is more associated with mechanical rigidity (Turgay et al., 2017; Nmezi et al., 2019).

Lamins are composed of an N-terminal domain, or head, a central α-helical rod and a globular C-terminal domain containing a nuclear localization signal (NLS), an Ig-fold domain and a CAAX-box prenylation motif (Gruenbam and Foisner, 2015; Dechat et al., 2008). These domains are implicated in membrane targeting and diverse contacts with multiple partners, including actin, nesprins, nucleoporins and histones (reviewed in Simon and Wilson, 2013). The central importance of lamins to correct cellular physiology is underscored by the plethora of lamin A mutations associated with heritable syndromes, known as laminopathies, most of which manifest as debilitating diseases (Kang et al., 2018).

Trypanosomes are protists of the Excavata supergroup, which separated from animals and their relatives over a billion years ago. The African trypanosome, *Trypanosoma brucei*, evolved a sophisticated strategy for establishing chronic infection in many mammalian hosts, which principally involves antigenic variation and mono-allelic expression of the superabundant variant surface glycoprotein (VSG). VSG is switched with sufficient frequency to facilitate a population continuing to infect the host (Mugnier et al., 2015; Pinger et al., 2017) despite robust host anti-VSG immune response (Stijlemans et al., 2016; Radwanska et al., 2018). For VSG switching to occur, monoallelic expression utilizes a dedicated transcriptional focus, the expression site body (ESB), together with telomeric silencing and silent VSG loci sequestered within heterochromatin (Figuereido and Cross, 2010). Hi-C analyses (Müller et al., 2018) highlight that subtelomeric regions bearing silent VSGs are folded into highly compact compartments with a high frequency of DNA–DNA contacts, likely important for maintaining a quiescent state. Significantly, monoallelic expression and VSG switching are both impacted by disruption of the trypanosome nuclear lamina (DuBois et al., 2012; Maishman et al., 2016), suggesting a role in regulating subtelomeric surface antigen expression.

There are two known components of the trypanosome lamina, NUP-1 and NUP-2. Both are essential, have predicted coiled-coil structure and have molecular masses of 450 kDa and 170 kDa, respectively. NUP-1 and NUP-2 localize to the NE periphery and have a clear structural role, as depletion leads to abnormalities in nuclear morphology and NPC positioning. In addition to operating in close cooperation with each other, NUP-1 and NUP-2 influence positioning of telomeres and chromosomes, suggesting roles in chromosome and chromatin organization. Significantly, this includes effects on developmentally regulated genes, since knockdown in the mammalian form leads to an increase in levels of both normally silent VSG and procyclin transcripts, with these latter regulated proteins normally only being expressed in the insect stage (DuBois et al., 2012; Maishman et al., 2016). NUP-1 and NUP-2 lack any lamin-related domains (Koreny and Field, 2016) and are substantially larger, suggesting a distinct architecture to the metazoan lamin system, even though it shares many functions.

Here, we exploited a set of NUP-1 deletion mutants to dissect the trypanosoma lamina *in vivo*, demonstrating that both terminal domains have crucial roles in lamina assembly. The interactions of different domains with partners such as NUP-2, NPC components and chromosomes suggest that NUP-1 termini constitute hubs in a lamina network with scaffolding properties.

## RESULTS

### NUP-1 domains have distinct spatial distribution

NUP-1 possesses distinct N- and C-terminal domains, separated by an extensive region of near perfect α-helical repeats (DuBois et al., 2012). If extended as an α-helix, each NUP-1 polypeptide can span over 400 nm and thus potentially contact much of the trypanosome nuclear volume (Field et al., 2012; DuBois et al., 2012). To monitor *in vivo* the distribution of NUP-1 domains, we chose to independently consider each domain in relation to each other. We tagged the N- and C-termini of NUP-1 with HA and GFP, respectively, and the repeat region was visualized with an in-house affinity-purified polyclonal antibody (DuBois et al., 2012).

**Fig. 1.**
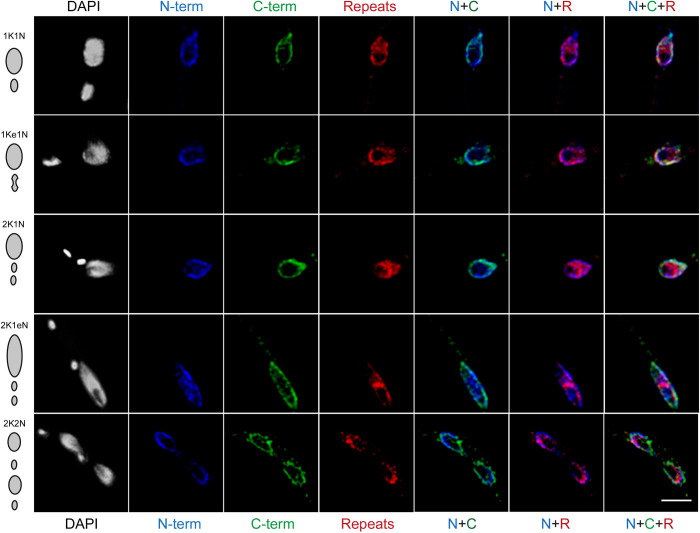
**NUP-1 domains relocate during the cell cycle.** Cells expressing a doubled-tagged version of NUP-1 were imaged by confocal microscopy. DAPI was used to visualize DNA (white). The N-terminal (N-term) and the C-terminal (C-term) domains were tagged with HA (blue) and GFP (green), respectively. To visualize the repeat region of NUP-1 (repeats), affinity-purified rabbit antibodies raised against the repeat were used (red). The typical distribution of NUP-1 at the nuclear periphery is clear throughout the cell cycle. Number of kinetoplasts and nucleus per cell across the cell cycle are depicted on the right (see text for details). Scale bar: 2 μm.

In African trypanosomes, the cell cycle stage can be assessed from the number and position of the nuclei and kinetoplasts (the latter a highly organized network of mitochondrial DNA). During interphase a single nucleus and kinetoplast (1K1N cells) are present, the latter becoming elongated (bilobed) during nuclear G_2_ phase (1Ke1N cells) to finally divide to produce cells with two kinetoplasts and only one nucleus (2K1N) prior to mitosis. After nuclear division, but prior to cytokinesis, cells with two nuclei are produced (2K2N) (Woodward and Gull, 1990; Benz et al., 2017). We found that both the N- and C-termini of NUP-1 were distributed similarly during interphase, but with distinct distributions in mitosis (Fig. 1). During inter and G_2_ phases both termini localized to the nuclear periphery. During mitosis, the N-termini accumulated in the nucleoplasm, while the C-termini localized at the periphery. At later stages, the N-termini were absent from the contractile ring in the NE formed in telophase, while the C-termini remained present across the nuclear periphery. NUP-1 terminal domains were also differentially located during mitosis and cytokinesis, which suggests that they may also have specific functions and independently engage with the machinery separating the two daughter nuclei. This behaviour likely reflects the flexibility/elasticity properties of NUP-1 as a coiled-coil and filamentous protein, with an ability to reposition during the cell cycle.

**Fig. 2.**
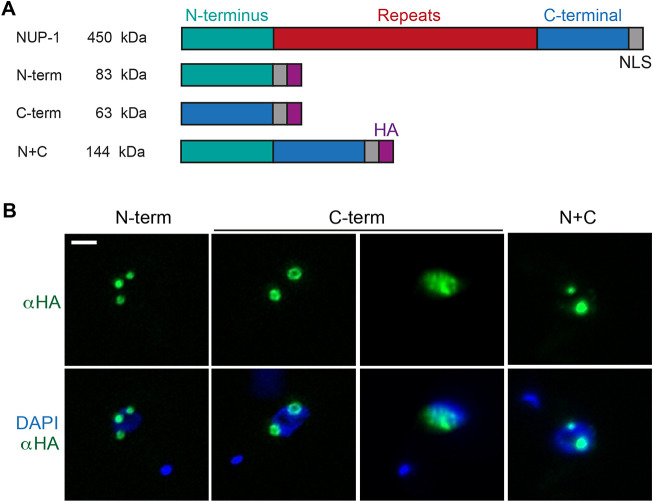
**NUP-1 terminal domains oligomerize.** (A) The fragments of NUP-1 designed for expression. WT NUP-1 is indicated at the top. All mutant sequences were cloned into pDEX-577, a Tet-inducible system expressing HA-tagged proteins. Protein sizes are indicated at left. The endogenous nuclear localization signal (NLS, grey) of NUP-1 was introduced into the N-terminal variant; in other constructs the endogenous NLS was present. The position of the HA-epitope is in purple. (B) Immunofluorescence analysis in bloodstream forms after 24 h of induction with 1.0 μg/ml of Tet. Cells were probed with an anti-HA antibody (green) showing round assemblies of expressed NUP-1 domains for the N-terminal (N-term), C-terminal (C-term) and a fusion of N and C terminal domains (N+C). Maxi-assemblies for all mutants are shown and, for the C-terminal mutant, the nucleoplasmic phenotype is also presented. DAPI was used to visualize DNA. Scale bar: 2 μm.

Moreover, we observed that the region constituted by α-helical coiled coil repeats (NUP-1R, Fig. 2A) also had a unique location throughout the cell cycle; repeats were present at both the nuclear rim but also had a presence within more internal nuclear regions and this latter location became most pronounced at late mitosis/anaphase (Fig. 1; Fig. S3A,B,E). This suggests a dynamic retraction of the repeat domain to the poles as the nucleus completes division, with the possibility that the α-helical repeats may interact with chromosomes at the 2K1N (early anaphase) stage, potentially being involved with their segregation to the daughter nuclei. This behaviour resembles that of the cohesins, which embrace sister chromatids from S-phase to anaphase. The NUP-1 repeat region shares high structural similarity with structural maintenance of chromosomes (SMC) proteins (Fig. S1), a superfamily of chromosomal DNA compaction proteins with DNA and ATPase activities, engaging in various processes of chromosome organization (Yatskevich et al., 2019). This does not exclude the possibility that the α-helical repeats of NUP-1 interact with other components of the mitotic machinery.

### The N- and C-terminal domains of NUP-1 assemble as organized structures

The specific folding of individual domains of mammalian lamins facilitates precise assembly and higher order structure. A recent study of the molecular architecture of mammalian lamin A, mapping interactions within lamin dimers and polymers recognizes that head, tail, linkers and rod domains all contribute differentially to the molecular architecture. For example, the linkers and head-tail regions are proposed to act as ‘springs’ contributing to the dynamic stretch and flexibility of lamin A, with multiple electrostatic interactions between adjacent rods and between head-to-tail and adjacent rods within a lamin dimer (Makarov et al., 2019).

To determine whether there are similar domains with specific functions present in NUP-1, we ectopically expressed the individual terminal domains (DuBois et al., 2012). Three constructs were created, encoding the N-terminal domain, the C-terminal domain and a truncation with the entire repeat region deleted (denoted N+C) (Fig. 2A). All constructs were validated by western blotting using an anti-HA antibody. The protein sizes for the N-terminal, C-terminal and N+C variants were 83 kDa, 61 kDa and 144 kDa, respectively. In the case of the C-terminal variant, a second band of ∼80 kDa was also detected, importantly, the presence of the 80 kDa band was clearly not in the parental line and was limited to tetracycline (Tet) induction conditions (Fig. S2A). We amplified and sequenced the tagged ectopic sequence of the C-terminal variant and confirmed that the construct transfected was as expected for the expression of the 61 kDa protein (data not shown). The reason for the presence of the ∼80 kDa HA-tagged C-terminal peptide is unknown, although it could be attributable to post-translational modifications, chromatin configuration or transcription utilizing alternate start or stop codons. Nevertheless, the exact cause for the slower migrating form is still unclear.

Following expression of all three domain constructs (N-terminal, C-terminal and N+C), circular ordered structures were assembled within nuclei (Fig. 2B) as evidenced by immunofluorescence. Interestingly, two distinct distributions were seen for the C-terminal, one forming assemblies and one with a diffuse interior nuclear localization (Fig. 2B; Fig. S2E). The round assemblies from the three constructs presented different sizes, and we named larger structures (Fig. 2B; Fig. S3) as maxi-assemblies (mean±s.d., 0.77±0.2 µm in diameter, range 0.5–1.7 µm) and the smaller structures (Fig. S3) as mini-assemblies (0.38±0.05 µm in diameter, range 0.23–0.45 µm), respectively (*n*=30 assemblies). Importantly, the diffuse nuclear pattern seen after C-terminal mutant expression is present concurrent only with mini-assemblies (Fig. S2E).

Moreover, the occurrence of assemblies was dependent on the concentration of Tet in the cultures and hence levels of protein produced, as well as the time of induction. We monitored the occurrence of assemblies with two different concentrations of Tet, 0.1 and 1.0 μg/ml, over 24 h (Fig. S2C,D). For the N-terminal construct induced at 0.1 μg/ml Tet, the number of assemblies remained low (mode=1 assembly) compared to inducing with 1 μg/ml Tet (mode=4 assemblies). For mini-assemblies induced with 0.1 μg/ml Tet, one to two assemblies were observed (range 1–10 per nucleus) whereas with 1.0 μg/ml Tet the number ranged from 1­–10 assemblies (Fig. S2C,D). For the C-terminal construct at low concentrations of Tet, the predominant phenotype was the diffuse nucleoplasmic pattern (frequency=0.8 in the population), and the frequency of assemblies in the population was low (<0.1 for maxi-assemblies, <0.02 for mini-assemblies). However, this proportion was reversed at high levels of Tet for maxi (mode=2, frequency 0.38), mini-assemblies (mode=1, frequency=0.12) and diffuse nucleoplasmic (frequency=0.26). As mentioned, mini-assemblies and the diffuse pattern can be found in the same nucleus (Fig. S2E), but not with maxi-assemblies. For the N+C construct, the frequency of assemblies with different concentrations of Tet remained similar (maxi and mini assemblies mode=1), with only a small difference in the range of assemblies per nucleus (Fig. S2C,D).

Furthermore, the time of induction also influences the number of assemblies per nucleus. We monitored the number of assemblies after induction with 1 μg/ml Tet at three different times, 12, 24 and 48 h (Fig. S2D). For the N-terminal variant, after 12 h, the number of assemblies ranged from one to seven (maxi) or up to 10 (mini), without a clear mode. After 24 and 48 h, the modes for maxi-assemblies were four and two, respectively (i.e. the number of assemblies was reduced by half). For the C-terminal mutant, one to two assemblies per nucleus was the most predominant phenotype, and this did not change drastically. By contrast the diffuse nucleoplasmic localization did change, with just 8% of the cells in the population having this phenotype at 12 h and gradually increasing to 36% after 48 h (Fig. S2D). In cells showing a nucleoplasmic localized C-terminal mutant, this distribution co-occurred with mini-assemblies in ∼30% of the cells in the population (Fig. S2E,F), as seen after 24 h of induction with 0.1 and 1.0 μg/ml Tet. For the N+C truncation, the localization was stable across the time [maxi-assemblies mode=1 (24 h), mode=2 (48 h)]. Moreover, maxi-assembly behaviour was monitored throughout the cell cycle (Fig. S3D). For all the constructs, the mode was one for assemblies in interphase (1K1N) and post-mitosis (2K2N), with a tendency at this stage to remain as low numbers. During G_2_ phase (1Ke1N) and mitosis (2K1N) a broader range of number of assemblies per nucleus appeared. We hypothesize that, as the cell prepares for cell division, it probably also divides assemblies for inheritance by daughter nuclei.

Our interpretation of these observations is that for the N-terminal and repeat deletion constructs, mini-assemblies mature into maxi-assemblies as the proteins accumulate. Additionally, this also suggests that assemblies gradually build, and are influenced by time and levels of protein in the nucleoplasm. The C-terminal domain seems more sensitive to these factors, as it can also be found with a nucleoplasm diffuse localization (Fig. 2B; Fig. S2E), with the possibility that this domain extends into the nucleoplasm during the cell cycle. This is consistent with previous observations showing that the C-terminal domain is not required for positioning of NUP-1 at the nuclear membrane (DuBois, et al., 2012). When combined with the N-terminal sequences, the capacity of the C-terminal to assemble is increased since the N+C domain variant is never seen as nucleoplasmic and its behaviour resembles that of the N-terminal variant. This indicates that the ability of the N-terminal domain to self-assemble is likely stronger than that of the C-terminus.

Similar nuclear assemblies obtained with mutant forms of lamins in metazoan cells have been reported (Yang et al., 2013; Sylvious et al., 2008; Hübner et al., 2006; Izumi et al., 2000). The mechanism for formation of these foci is not well understood, but in some cases they are related to disease mutants that disrupt assembly and hence function. Interestingly, not all lamin mutants result in nuclear aggregates (Sylvious et al., 2008 and references therein). Although the mechanism of formation of assemblies from NUP-1 terminal domains is also unclear, the self-assembly properties of the N- and C-terminal domains is very pronounced. Furthermore, no obvious defects to overall cell morphology were observed, although a small increase in cell cycle time in a Tet dose-dependent manner was observed in induced cultures followed for up to 6 days (Fig. S2B). Thus, although there is a detrimental effect in terms of replication rate, indicating a loss of fitness, the presence of these NUP-1 assemblies is non-lethal, at least in the short term, as is the case for many lamin mutants (Sylvius et al., 2008; Muchir et al., 2003, 2004; Mounkes et al., 2005; Stuurman et al., 1999).

### NUP-1 domains disrupt endogenous NUP-1 localization

**Fig. 3.**
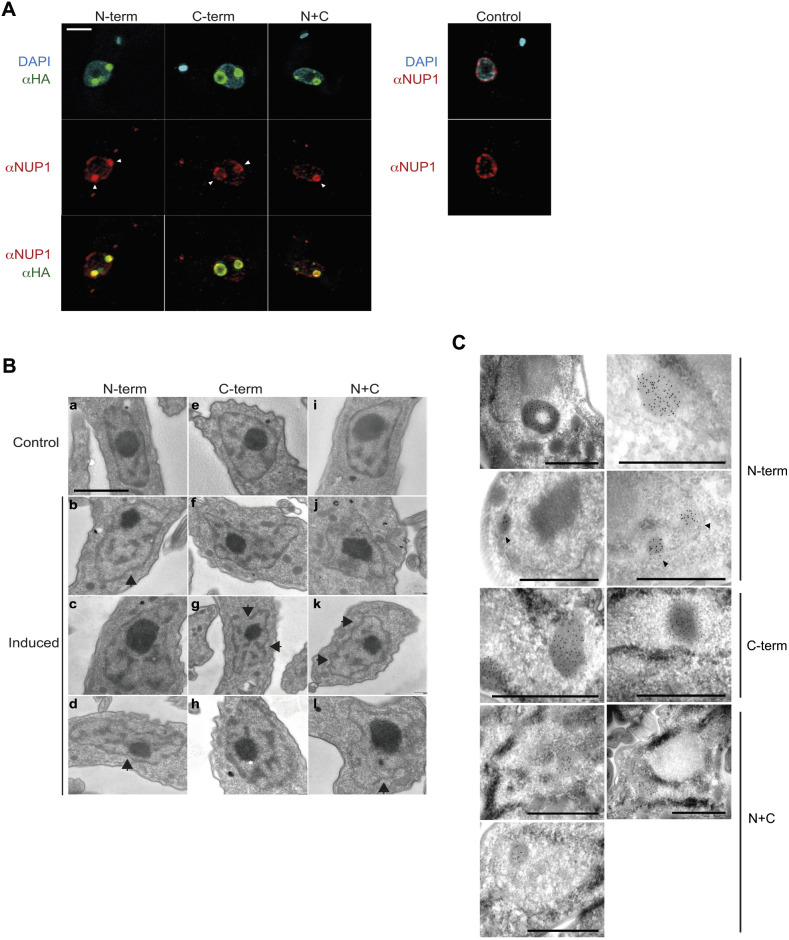
**NUP-1 domains interact with and disrupt normal arrangement of endogenous lamina proteins.** BSF cells containing the NUP-1 domain constructs were fixed, stained and visualized by either confocal immunofluorescence microscopy (A) or electron microscopy (B,C). Overexpression of NUP-1 domains was induced with 1.0 μg/ml of Tet for 24 h. (A) The normal NE arrangement of endogenous NUP-1 (control) is disrupted by the expression of NUP-1 N-term, C-term and N+C domain constructs. Cells were co-stained with anti-HA (green) and anti-NUP-1 repeat antibodies (red), and DAPI (cyan), as indicated. Central *z*-stacks are presented. Arrowheads highlight NUP-1 domain assemblies. Scale bar: 2 μm. (B) Disruption of the normal structure of the nuclear membrane visualized by electron microscopy after expression of the N-term (b–d), C-term (f–h) domains and the N+C fusion (j–l). Respective control cells without induction are shown (a, e and i). Disruption is shown as irregular edges in the nuclear membrane. Arrows highlight examples of irregular nuclear membrane boundaries in panels b, d, g, k, l. Scale bar: 1 μm. (C) Immunogold localization of NUP-1 mutant variants (N-, C-terminal and N+C fusion). Gold particles are detected in the assemblies of NUP-1 domain constructs, which also appear as electron dense. Black arrowheads show foci of NUP-1 assemblies in proximity to the nuclear membrane. Scale bars: 1 μm.

To determine in more detail the impact of NUP-1 fragments upon lamina organization, we performed immunofluorescence using the NUP-1 α-helical repeat antibody to visualize the endogenous NUP-1 protein in the presence of the domain constructs. All three NUP-1 domain constructs colocalized with NUP-1 coiled-coil repeats and partially disrupted the nuclear peripheral distribution of NUP-1 (Fig. 3A). Importantly, although assemblies were stable across the cell cycle, associations between NUP-1-domain constructs and NUP-1 repeats (NUP-1R) from the endogenous NUP-1 were seen to be favoured during interphase (Fig. 3A; Fig. S3A–C).

Label-free mass spectrometry of whole-cell lysates indicated that expression levels of endogenous NUP-1 in the N-terminal and N+C domain-expressing cells were essentially unaltered compared to what was seen in wild-type cells (ratios 0.82±0.04 and 0.97±0.08 vs wild type, mean±s.d., respectively) and hence that endogenous protein is recruited to NUP-1 assemblies. By contrast, C-terminal domain-expressing cells accumulated more endogenous NUP-1 than wild-type cells (ratio 2.21±0.47 vs wild type), without significantly affecting proliferation (Fig. S2B), indicating that a modest excess of NUP-1 is tolerated.

With endogenous NUP-1 being recruited to the assemblies, we asked whether sequestering NUP-1 impacted NE integrity. Cells expressing NUP-1 domain constructs possessed an altered nuclear membrane morphology (Fig. 3B) with irregular boundaries (81%, 86% and 83% for N-terminal, C-terminal and N+C terminal variants, respectively, *n*≥18 cells), consistent with altered/modified lamina support (percentage of cells with detectable irregular nuclear boundaries in control cells is 10%, *n*=70 cells).

We corroborated the presence of NUP-1 domain constructs by immunogold electron microscopy. We confirmed the presence of well-defined gold-labelled high-density circular structures inside the trypanosome nucleus in Tet-induced cells (Fig. 3C), correlating with the immunofluorescence observations (Figs 2B and 3A). These structures were frequently associated with the NE (Fig. 3C, black arrowheads), supporting our evidence that the constructs interact with endogenous NUP-1 (Fig. 3A) and possibly additional components of the nuclear membrane (see Figs 6 and 7A).

Furthermore, it is known that NUP-1 and NUP-2 are associated with a repressive heterochromatin environment and regulating expression of VSG genes (DuBois et al., 2012; Maishman et al., 2016), which normally organized into heterochromatin when in a quiescent state (Figuereido and Cross, 2010). Importantly, there was a normal retention of heterochromatin observed as electron-dense regions (Fig. 3B,C) indicating no major disruption to heterochromatic regions. This is consistent with transcriptome and proteome analyses (Fig. S5, Table S1), which provided no evidence for disruption to parental VSG 427-3 (alias VSG 224) expression or other VSG genes. Overall, these data suggest that heterochromatin, monoallelic expression and VSG switching are preserved in the subtelomeric VSG loci during expression of NUP-1 domain constructs.

### NUP-1 domain localizations with chromatin and telomeres

**Fig. 4.**
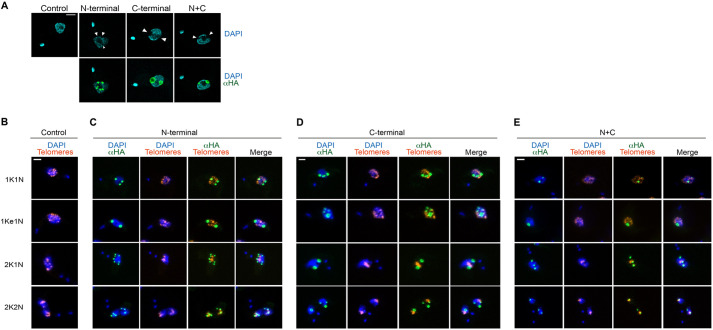
**NUP-1 domains co-occur with chromatin without disruption.** Cells bearing NUP-1 variants tagged with HA were fixed, stained as indicated and visualized by confocal immunofluorescence microscopy with a Leica System (A) or Zeiss system (B–E). In all cases, overexpression of NUP-1 domains was achieved using 1.0 μg/ml of Tet during 24 h. (A) Chromatin is displaced by the overexpression of the three NUP-1 variants. Arrowheads show the void of DNA created by presence of NUP-1 assemblies. SMB cells lacking the overexpression system are used as control. Cells are stained with anti-HA antibody (green) and DAPI is used to visualize DNA (cyan). (B–E) FISH assay. Cells expressing the NUP-1 mutants were used for FISH assay, using a telomere-pairing probe (orange) and an anti-HA antibody to recognize NUP-1 variants (green). DAPI was used to visualize DNA (blue). (B) Control SMB cells displaying the normal telomere arrangement. Interaction of telomeres with the overexpression mutants are shown in (C) N-terminal, (D) C-terminal and (E) N+C mutants. Central *z*-stacks are shown. All scale bars: 2 μm.

The nuclear lamina controls gene expression by modulating chromatin organization, a mechanism common to all known lamina systems (Gruenbam and Foisner, 2015; Dechat et al., 2009; Koreny and Field, 2016). Expression of NUP-1 domain constructs led to voids in DNA as observed by DAPI staining and revealed by super-resolution immunofluorescence (Fig. 4A). This alteration of DNA distribution was observed for all three NUP-1 fragments and the phenomenon may also contribute to the disturbed morphology of the NE (Fig. 3B). It is most likely that this is a physical phenomenon, whereby the DNA is simply excluded from dense NUP-1 domain regions, and presumably the free energy of NUP-1 domain self-assembly is sufficient to exclude chromatin. Similar voids in chromatin distributions have also been reported to occur in COS7 cells with a lamin A mutation (Q432X) (Yang et al., 2013), which is a mutation that is also known to lead to cardiac disease (Yang et al., 2013; Sylvius et al., 2008), although the mechanisms causing such voids in the DNA distribution in two different models (COS7 cells and trypanosomes) while expressing mutated versions of a lamina protein lacks clarity.

Given evidence that NUP-1 modulates positioning and movement of the telomeres (DuBois et al., 2012; Field et al., 2012), we performed fluorescence *in situ* hybridization (FISH) to obtain evidence for targeting of NUP-1 terminal domains to telomeres (Fig. 4B–E). We did not detect a strong association between any of the NUP-1 constructs and telomeres across the cell cycle. Nevertheless, during mid- and late mitosis, telomeres (compacted and aligned in the centre of the nucleus) approach NUP-1 assemblies and occasionally occur in the same nuclear foci, yet, no evidence of significant interaction between these nuclear structures was detected and co-occurrence may simply represent segregation of telomeres and NUP-1 termini into the daughter nuclei. In spite of the presence of NUP-1 assemblies, telomeres segregate normally, consistent with cells being tolerant to the presence of the assemblies during several days. Moreover, during mitosis, assemblies also migrate towards opposite poles of the nucleus (Fig. 4B; Fig. S3A–C).

### NUP-1 interacts with specific NPC components

In the mammalian bloodstream trypanosome, multiple mechanisms ensure mono-allelic expression of a single VSG from a telomeric expression site (Mugnier et al., 2015; Faria et al., 2019; Glover et al., 2016; Saura et al., 2019). In some insect stages, the VSG coat is replaced by procyclin (Roditi et al., 1989), and similar to VSG, procyclin genes are transcribed by RNA Pol I, but from chromosomal internal sites rather than a telomere. Importantly, NUP-1 participates in silencing of both VSG and procyclin genes (DuBois et al., 2012). With both N-terminal and C-terminal domains occasionally coincident with telomeres, we asked whether these constructs triggered alterations in the global proteome, and undertook unbiased, label-free mass spectrometry of whole-cell lysates to address this.

**Fig. 5.**
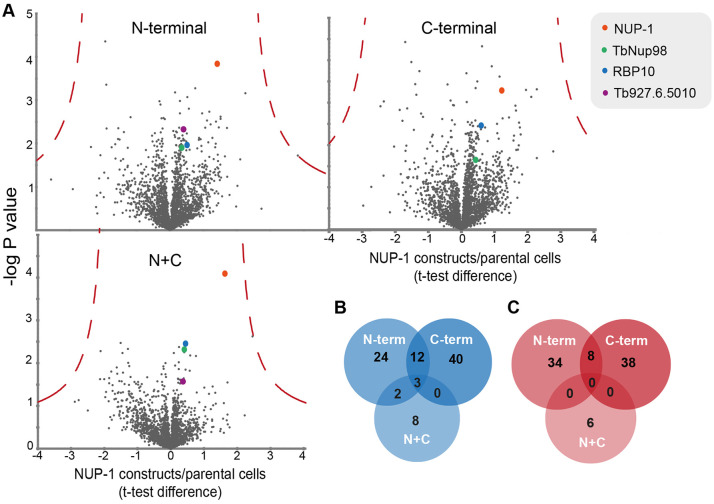
**NUP-1 domain constructs lead to specific changes to the proteome.** (A) Volcano plots of protein abundance changes in cells expressing N-terminal, C-terminal and N+C constructs from three replicates. Cells expressing the N-, C-terminal and N+C mutants were induced with Tet (1.0 μg/ml) for 24 h. Statistical differences (as compared to parental SMB cells) are plotted against −log *P* value. An FDR of 1% is shown as a dotted red line. NUP-1 is the orange dot, together with TbNup98 (green), RBP10 (blue) and protein Tb927.6.5010 (purple). (B,C) Venn diagrams show differentially expressed proteins, either (B) upregulated or (C) downregulated in cells expressing NUP-1 variants.

Over 2500 protein groups were identified and quantified (Fig. 5; Table S1). For selection of differentially expressed proteins, we applied the following inclusion filters: (1) at least two unique peptides identified, (2) ratio >±0.2, (3) statistical significance (log *P*)>1.5 −log *P*-value and (4) *t*-test difference of ±0.3 with respect to control cells. Following filtering, 83, 101 and 19 differentially expressed protein groups were detected for cells expressing N-terminal, C-terminal and N+C domains, respectively (Fig. 5), and which corresponds to 1%, 1.2% and 0.23% of the predicted proteome (Aslett et al., 2010). Therefore, there is minimal overall impact on the proteome upon expression of NUP-1 constructs. The false discovery rate (FDR) was calculated (FDR=0.01, s0=2), and although none of the proteins met this cut off, some differentially expressed proteins (proteins with changes in their corresponding expression levels) showed consistency in all three replicates.

As expected, peptides corresponding to NUP-1 were considerably more abundant in all three cell lines. Ratios for NUP-1 termini versus control cells were 6.33±2.18, 3.75±3.25 and 4.67±0.99 (mean±s.d.) for N-, C- and N+C terminal constructs, respectively. As described above, compensatory upregulation of endogenous NUP-1 is only observed for the C-terminal variant.

Furthermore, only the nucleoporin TbNup98 (Tb927.3.3180) and RNA-binding protein 10 (RBP10) (Mugo and Clayton, 2017) were upregulated in all three domain cell lines (Table 1; Table S1). TbNup98 is a PheGly (FG) nucleoporin component of the NPC and likely restricted to kinetoplastids (Obado et al., 2016). RBP10 is an RNA-binding protein that functions as a major regulator of development (Mugo and Clayton, 2017).

**Table 1.**
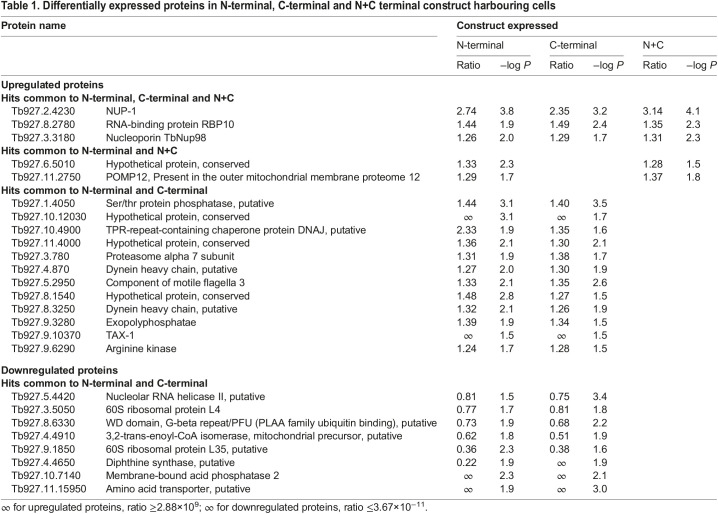
Differentially expressed proteins in N-terminal, C-terminal and N+C terminal construct harbouring cells

Among proteins upregulated in cells expressing N-terminal domains were an mRNA-binding protein (Tb927.6.5010) and Tb927.11.2750 (Table 1; Table S2). The Tb927.6.5010 gene product corresponds to a potential mRNA fate regulator, acting as a post-transcriptional repressor (Lueong et al., 2016; Erben et al., 2014; Goos et al., 2017). The gene product of Tb927.11.2750 was also upregulated and is restricted to *T. brucei*, *T. gambiense*, *T. evansi* and *T. cruzi*. Furthermore, downregulated proteins were also detected (Fig. 5C; Table 1, Table S3). Eight such proteins were quantified with high confidence in both the N-terminal and C-terminal domain (Table 1). This list includes an RNA helicase (Florini et al., 2019) and diphtine synthase (Aslett et al., 2010), which has been implicated in different aspects of RNA metabolism and localized in the nucleus (Dean et al., 2017). Tb927.11.15950, another downregulated protein, is annotated as a transporter (Aslett et al., 2010), and recognized by BLAST to have analogy with nucleobase transporters. Moreover, Tb927.11.15950 shows partial nuclear localization (Dean et al., 2017). These results suggest that the NUP-1 domain constructs may attenuate nuclear RNA processing.

With these changes at proteome level, we performed RNA-seq to detect potential transcriptome alterations as a result of overexpression of NUP-1 domains. No evidence for differential expression was found for any transcript (Fig. S5), and the abundance of VSG and procyclin mRNA were unaltered. Similarly, proteomics revealed no modification in VSG expression compared to the parental cell line. Although originally expressing VSG 427-2 (Tb427.BES40.22) (Wirtz et al., 1999), we uncovered a switch in the parental cell line to VSG 427-3 (Tb427.BES65.13) but beyond this, no alterations were detected.

### TbNup98 interacts with both N-terminal and C-terminal domains of NUP-1

**Fig. 6.**
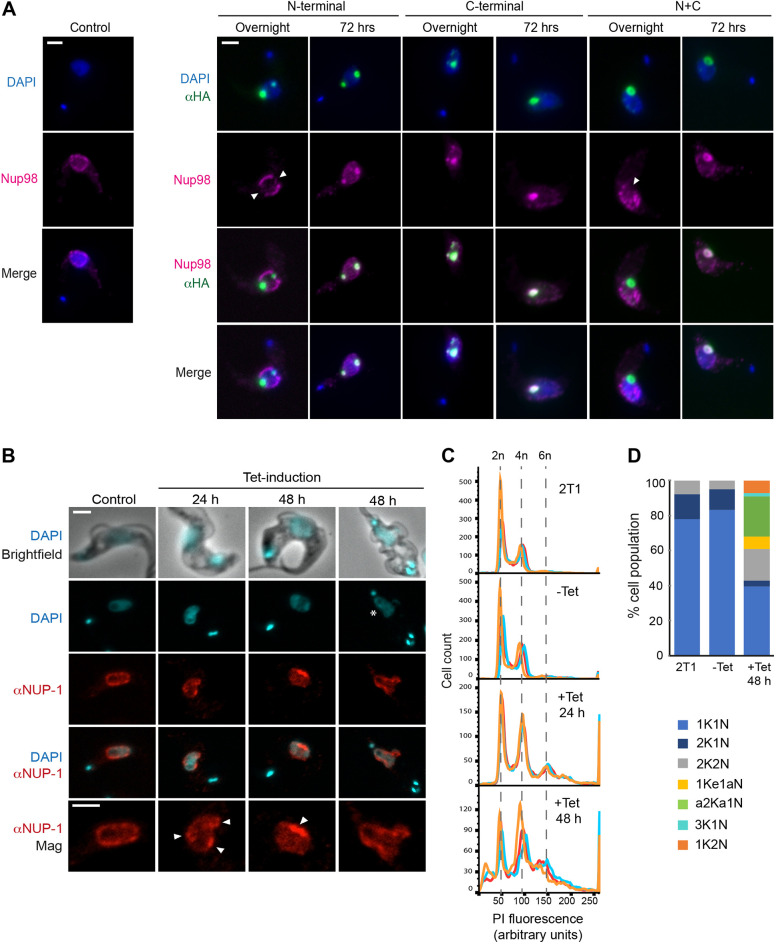
**NUP-1 depends on TbNup98 to maintain NE structure.** (A) TbNup98 and Tet-induced (1.0 μg/ml) cells bearing NUP-1 constructs were visualized after 16 (overnight) and 72 h by confocal immunofluorescence microscopy. Uninduced cells are used as control to visualize the distribution of TbNup98. After overnight induction, there are regions lacking TbNup98 normal distribution (arrowheads) in the N-terminal and N+C mutants whereas after 72 h, all NUP-1 variants are coincident with TbNup98. Central *z*-stacks are shown. Scale bars: 2 μm. (B) The impact of TbNup98 knockdown on normal NUP-1 distribution was followed at 24 h and 48 h. Cells were fixed, stained and visualized by confocal immunofluorescence microscopy. Cells are co-stained for NUP-1 repeat (red) and DAPI (blue), as indicated. After induction, NUP-1 starts to localize in foci (arrowheads). After 48 h, ‘monster cells’ start to appear. Abnormal clustering of NUP-1 distribution is seen (arrowheads) as well as aberrant DNA containing-bodies (white star). Central *z*-stacks are shown. Scale bars: 2 μm. (C) Flow cytometry analysis of DNA content in TbNup98 depleted cells. Histograms indicating number of cells versus propidium iodide (PI) fluorescence. The analysis was conducted for control cells (2T1 cells), uninduced cells and Tet-induced cells at 24 and 48 h. Histograms represent three independent experiments, represented by orange, blue and red lines. Peaks labelled 2n represent diploid cells; 4n, tetraploid cells and 6n/8n, higher ploidy cells. Flow cytometry profiles for 10,000 propidium iodide-labelled cells are shown. (D) Cell cycle progression after TbNup98 silencing followed by microscopy. 2T1, uninduced and induced RNAi cells (*n*=100 cells each) were fixed and stained with DAPI. Normal 1K1N, 1Ke1N, 2K1N and 2K2N cells were detected. In the population of induced cells, a series of aneuploid cells were observed: 1Ke1aN (abnormal nuclei), a2Ka1N (cells in 2K1N with abnormal kinetoplasts attached to amorphous nuclei), 3K1N, 1K2N.

TbNup98 has multiple connections to different regions of the NPC, including other FG Nups, the nuclear basket and the inner and outer rings (Obado, et al., 2016). As proteomics suggested a connection between NUP-1 and TbNup98, we analysed the locations of NUP-1 domain constructs and TbNup98. As previously reported (DuBois et al., 2012; DeGrasse et al., 2009), TbNup98 clearly appeared as punctua at the NE, consistent with an NPC association (Fig. 6A). After overnight induction, TbNup98 colocalized with the C-terminal assemblies, but not the N-terminal and N+C assemblies. In these latter cases, where these constructs were located there was a zone depleted of nuclear pore complexes (Fig. 6A; Fig. S6). However, after 72 h induction, TbNup98 colocalized with all three NUP-1 domain constructs and the associations were retained across the cell cycle, supporting a potential interaction. This suggests that, although both NUP-1 termini can interact with TbNup98, the C-terminus is more likely to support such interactions.

### NUP-1 depends on TbNup98 to maintain NE structure

With proteomics and immunofluorescence analysis suggesting an interaction between the NUP-1 termini and TbNup98, we decided to explore the relationship further by gene silencing. After 24 h of TbNup98 knockdown the classical distribution of NUP-1 at the nuclear periphery was lost, and the protein instead clustered at specific points of the NE. Moreover, TbNup98-depleted cells possessed nuclei with an altered morphology, including blebs and protuberances (Fig. 6B). After 48 h induction, NUP-1 clustering became more significant in aberrant TbNup98-depleted nuclei (Fig. 6B, arrowheads). Significantly, ‘monster’ cells appeared, with evident damage to nuclear shape and aberrant foci of chromatin separated from the nucleus (Fig. 6B, white star). The ploidy of cells was altered following TbNup98 depletion as detected by flow cytometry, with a gradual decrease in diploid cells and an increase in tetraploid and higher polyploid (>4n) cells in the population (Fig. 6C). This suggests that DNA duplication takes place, but cells are incapable of completing mitosis and cytokinesis. Moreover, TbNup98 knockdown cells exhibited abnormal DNA-containing bodies, with defects in the segregation, shape and number of kinetoplasts and nuclei (Fig. 6D) as detected by microscopy analysis (*n*=100 cells). In particular, 2K1N cells bearing extra structures containing chromatin were prevalent among these abnormal cells. These results indicate that TbNup98, apart from its function as part of the NPC, has an influence on mitosis, cytokinesis and/or normal segregation of chromatin and participates with NUP-1 to maintain NE integrity.

### The lamina protein NUP-2 mainly interacts with the NUP-1 N-terminus

**Fig. 7.**
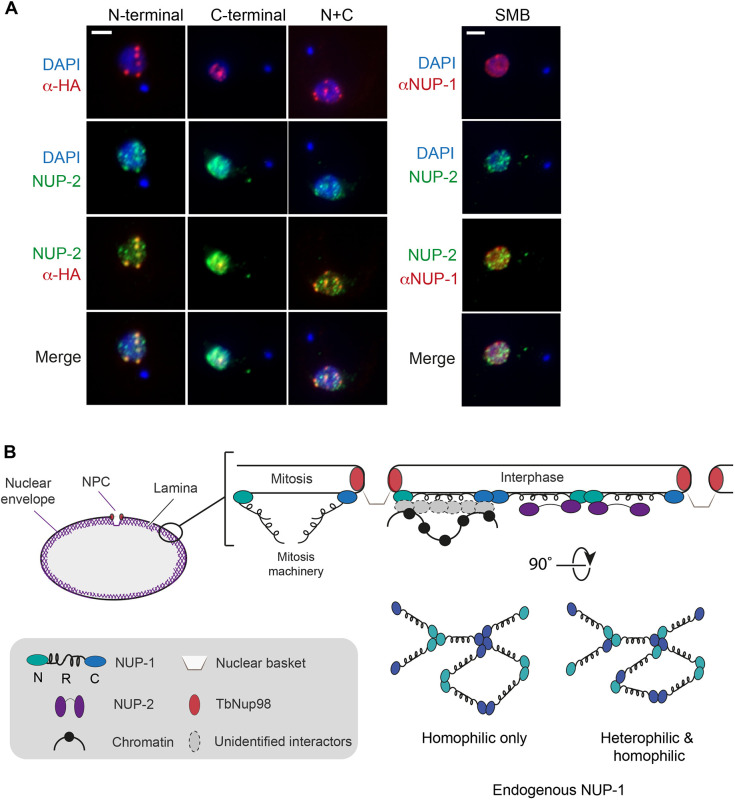
**NUP-2 interacts with the N-terminal domain of NUP-1.** (A) Parental SMB cells expressing NUP-2::TY1 were co-stained with anti-TY1 antibodies (green) and anti-NUP-1 repeat antibodies (red). Cells expressing NUP-2::TY-1 and domain constructs were co-stained with anti-HA (red) and anti-TY1 (green) antibodies. NUP-2::TY1 colocalizes with N-term::HA and N+C::HA. DAPI was used to visualize DNA (blue). Central *z*-stacks are shown. Scale bars: 2 μm. (B) Model for interactions between NE proteins within the trypanosoma lamina. NUP-1 may form interactions that are head-to-head, tail-to-tail (homophilic interactions) and head-to-tail (heterophilic interactions). NUP-1, via the N-terminal domain, interacts with NUP-2, while both of the NUP-1 terminal domains contact TbNup98 in the NPC. NUP-1 regulates expression of VSG and procyclin genes, although the exact mechanism is still unknown. During mitosis, the NUP-1 repeats (R) localize in the nucleoplasm, suggesting participation/interaction with mitotic machinery. See text for further details.

NUP-2 is the second defined component of the trypanosome lamina. NUP-1 and NUP-2 are intimate interactors and cooperate to maintain NE architecture (Maishman et al., 2016). To better understand interactions between NUP-2 and NUP-1, we used a cell line co-expressing a TY1-tagged version of NUP-2 together with NUP-1 domain constructs. We observed the canonical distribution of NUP-2 across the nuclear periphery in wild-type cells (Fig. 7A). In cells expressing NUP-1 mutants, NUP-2 clearly associated and colocalized with N-terminal and N+C variants, forming specific foci (Fig. 7A). Interestingly, NUP-2 was not found to colocalize strongly with the C-terminal domain or endogenous NUP-1 repeats. Importantly, all these interactions were maintained across the cell cycle (Fig. S7), suggesting that NUP-2 mainly interacts with the N-terminal domain of NUP-1.

## DISCUSSION

Several lamina systems are now known, specifically the ‘metazoan’ lamins, nuclear matrix constituent proteins (NMCPs) in plants and NUP-1 and -2 in kinetoplastids (Koreny and Field, 2016). Lamins assemble to form fibrils with a precise architecture governed by interactions between specific domains, with parallel dimers assembling in antiparallel fashion (Ahn et al., 2019; Makarov et al., 2019) and that are anchored to the NE via C-terminal prenylation. Lamin B is likely capable of supporting lamina functions alone, a conclusion supported by both expression profiles and phylogenetics (Ahn et al., 2019; Koreny and Field, 2016), while both A and B lamins can interact directly with core histones with roles in the formation of lamina-associated domains.

By contrast to A and B lamins, NUP-1 is an elongated protein, spanning a significant fraction of the nuclear volume, is highly flexible with distinct domains targeted differentially and likely presenting intra- and inter-molecular interactions via both termini (Fig. 7B). Whether NUP-1 oligomerizes via the extensive coiled-coil repeat region is unknown, but this is a common property of coiled-coil proteins (Lupas and Gruber, 2005). The observation that telomeric regions have restricted contacts with other genomic regions in trypanosomes is also consistent with the positioning of NUP-1 and the wild-type termini. Partitioning is a common mechanism for nuclear subdomains, and significantly allows exchange of VSG expression sites from heterochromatin NUP-1 into the expression site body. If these structures resemble membrane-less condensates due to self-assembly remains to be demonstrated.

NUP-1 localizes at the nuclear periphery during interphase with the repeat region migrating into the nuclear interior during mitosis, potentially contributing to chromosome segregation and engaging with the mitotic machinery – the repeat has some sequence similarity to SMC proteins and an interaction between NUP-1 and KKIP1, a kinetochore protein, has been demonstrated (D'Archivio and Wickstead, 2017). SMC proteins, which make multiple contacts with DNA at telomeres, centromeres and chromosome arms are present in trypanosomes (Gluenz et al., 2008; Bessat and Ersfeld, 2009). Furthermore, both the trypanosome-type kinetochore and NUP-1 and NUP-2 are present throughout the Kinetoplastida, but not in relatives of the lineage, e.g. *Euglena gracilis*, which is entirely consistent with a functional connection between NUP-1 and the kinetochore. Significantly, participation of NUP-1 in chromosome segregation may also explain how the very large number of chromosomes are segregated in the trypanosome nucleus in the absence of a highly complex microtubule array within the spindle (Ersfeld and Gull, 1997; Ogbadoyi et al., 2000).

Both N- and C-terminal domains of NUP-1 assemble as circular assemblies that mature into larger structures capable of recruiting endogenous NUP-1. These structures come to lie close to the NE in most cases, and present a somewhat homogenous structure. In mammalian cells, similar structures have been reported to form inside the nucleus as a response to specific mutations in lamin A (Yang et al., 2013; Muchir et al., 2004; Hübner et al., 2006). Moreover, although both N-terminal and C-terminal domains contain coiled-coil regions, they exhibit distinct properties, with the C-terminal having less-efficient self-association and displaying a nucleoplasmic phenotype, suggesting that a fraction of the C-terminal domain of NUP-1 may shift into the nucleoplasm. Two phenotypes can be seen with the C-terminal mutant (circular assemblies and nucleoplasmic form) and further data are required to fully understand the reason for the presence of both phenotypes, as well as for the slower migrating form of the HA-tagged protein. Interestingly, a nucleoplasmic subfraction of lamin A has been implicated in regulating proliferation, differentiation, cell cycle progression and interaction with euchromatin regions (Naetar et al., 2017; Vidak et al., 2018; Gesson et al., 2014) and whether a nucleoplasmic fraction of NUP-1 may exist and have similar funtions is still unknown. The NUP-1 N-terminus contains a NUP-2-binding site, but little overlap was detected between NUP-2 and endogenous NUP-1 coiled-coil repeats and therefore these two proteins potentially form separate meshworks connected by hubs within the NUP-1 N-terminal domain.

Proteomics revealed several interactions between NUP-1 and additional nuclear factors. Two mRNA-binding proteins, Tb927.6.5010 and RBP10, were differentially expressed upon induction of assemblies (Table 1). Tb927.6.5010 is a potential post-transcriptional repressor (Lueong et al., 2016; Erben et al., 2014) whereas RBP10 is a regulator of developmental expression and promotes progression from the procyclic form to bloodstream form. RBP10 binds to a 3′-UTR motif in procyclic-specific mRNAs, targeting them for translational repression and degradation (Mugo and Clayton, 2017). Lamins also interact with RNA-binding (Siddam et al., 2018), RNA-processing (e.g. splicing machinery) and RNA transport proteins (Zahr and Jaalouk, 2018; Depreux et al., 2015). An example is the RNA-binding protein Celf1, which participates in a cascade involving kinases required for normal phosphorylation of lamin A/C (Siddam et al., 2018). With RBP10 also capable of recruiting kinases (Mugo and Clayton, 2017) it is likely that an analogous mechanism operates in *T. brucei*.

Levels of TbNup98, an FG nucleoporin and component of the trypanosome NPC (Obado et al., 2016; DeGrasse et al., 2009), were also altered in the NUP-1 domain-expressing cells. Importantly, TbNup98 has an established physical interaction with NUP-1 and NUP-2 by co-immunoprecipitation (Obado et al., 2016), which is fully consistent with the data here. Importantly, there is an increase of TbNup98 in these cells expressing domain constructs, suggesting a compensatory mechanism for sequestration by NUP-1 domains. Significantly, not all the FG nucleoporins (FG nups) are essential for transport (Strawn et al., 2004), suggesting a role in other NE activities for these NPC components, some of which have already been described to influence mitotic chromosome dynamics and spindle assembly (Wu et al., 2016). For TbNup98, a role in mitosis and/or cytokinesis is possible as those activities are impaired after silencing. Interestingly, KKIP1 co-purifies with NUP-1 and some components of the NPC (D'Archivio and Wickstead, 2017), including TbNup92, which interacts with spindle poles during mitosis and with centromeres, contributing to the distribution of chromosomes during cell division (Holden et al., 2014). Silencing TbNup98 led to NUP-1 clustering and loss of the NE localization, and suggests that TbNup98 is a component of the NPC-mediated anchoring mechanism. Moreover, the abnormal ploidy and nuclear morphology with a failure to complete mitosis in TbNup98-silenced cells is consistent with a role in anchoring NUP-1 and consequent disruption of chromosome segregation. The influence of NPC components on mitosis in other eukaryotes has been already described, proving that the nucleoporins are essential for maintaining the associations of the kinetochores to microtubules, and for promoting spindle assembly and mitotic progression (Ibarra and Hetzer, 2015; Chatel and Fahrenkrog, 2011). Significantly, despite divergent sequence and origins of many components between trypanosomes and metazoan organisms, these comparisons suggest a convergence and retention of overall mechanistic similarity.

In summary, we propose a hub-and-spoke model for NUP-1 assembly (Fig. 7B) within the trypanosoma lamina. As NUP-1 termini can oligomerize, interactions may be occurring in a head-to-head, tail-to-tail or head-to-tail manner through co-occurring homophilic and heterophilic interactions. Furthermore, as terminal domains can recruit the repeats region, a sliding mechanism similar to that reported for lamin A filaments (Makarov et al., 2019) between NUP-1 molecules may be possible. Moreover, in the interaction with NUP-2, the N-terminal domain constitutes the main anchor point, providing additional stability. Additionally, both NUP-1 termini contact nucleoporin TbNup98 in the NPC, with the possibility that other components of the NPC can be contacted by NUP-1. During cell division, the NUP-1 α-helical coiled coil repeats localize to the nucleoplasm, suggesting (1) re-location from the NE and (2) participation/interaction with mitotic machinery. These will require further examination to fully understand the potential role of this trypanosoma lamin in mitosis, a case of closed cell division. Importantly, NUP-1 previously showed participation in the regulation of VSG and procyclin genes, pathogenesis-related genes (DuBois et al., 2012; Maishman et al., 2016), although the mechanism and potential partners await discovery.

## MATERIALS AND METHODS

### Cell culture

Bloodstream form *Trypanosoma brucei brucei* Lister 427 were cultured as previously described (Hirumi and Hirumi, 1989) in HMI-9 medium. Single marker bloodstream form (SMB) and 2T1 bloodstream form (Lister 427, MITat1.2, clone 221a) cells were used for expression of tetracycline-inducible systems, pDEX (Kelly et al., 2007) and RNA interference (RNAi) (Alibu et al., 2005; Alsford and Horn, 2008), respectively. 2T1 cells were maintained in medium containing phleomycin and puromycin (1 μg/ml and 0.5 μg/ml, respectively). When antibiotic selection was required, drugs were used at the following concentrations: phleomycin 2.5 μg/ml, hygromycin 5 μg/ml and blasticidin 5 μg/ml.

### Recombinant DNA manipulations

Different regions of the NUP-1 coding sequence were HA-tagged in the pDEX-577G vector, a tetracycline-inducible system (Kelly et al., 2007). A modified version of pDEX-577G was used changing the GFP-tag for HA. The inserts were introduced into BamHI and HindIII sites. The regions of NUP-1 used to build the overexpression variants are shown in Fig. S8. The constructs were linearized with NotI and used for transfection of SMB cells.

### Transfection of bloodstream form cells

Approx. 10 μg DNA was used for every 2×10^7^ cells transfected. Usually, 1.5–3×10^7^ cells were electroporated in either Cytomix (Ngô et al., 1998; Burkard et al., 2007) or Tb-BSF buffer (Schumann-Burkard et al., 2011) using an Amaxa Nucleofactor II, program X-001 (Lonza Bioscience). Positive clones were assayed for correct insertion and expression of desired protein by immunofluorescence and immunoblot.

### Western blotting

5×10^6^ cells were resolved by 4–12% SDS-PAGE (Invitrogen). Proteins were transferred to a PDVF membrane (Millipore). An anti-HA mouse antibody (mouse, Santa Cruz Biotechnology 7392) was used at 1:3000. Detection with secondary anti-mouse IgG peroxidase (Sigma A9044) was performed at a dilution of 1:8000. Visualization was made by chemiluminescence with ECL-detection reagents (GE Amersham RPN 2106). Images were captured using X-ray film (GE Amersham 28906837).

### Proliferation analysis

Cell cultures were adjusted to 10^5^ cells/ml. If required, cells were induced with tetracycline (Tet) in the culture medium. Cell numbers were determined using a Z1 Coulter counter every 24 h and diluted to 10^5^ cells/ml. All determinations were performed using triplicate cultures.

### *In situ* tagging

The pMOTag43M vector system (Oberholzer et al., 2006) was used to introduce a C-terminal *in situ* myc-epitope to TbNup98. The pPOT system (Dean et al., 2015) was used to introduce a N-terminal *in situ* TY1-epitope to Nup-2. The primer sequences are: TbNup98F, 5′-TGGGAATGCTTCAGCAAGTGGTGAAAAGAACAATGCTCCACGGAATCCCTTCTCATTTGGTGCCTCTTCTGGGAATGCTGGTACCGGGCCCCCCCTCGAG-3′; TbNup98R, 5′-ACTAAAGAAGGGTAGAAAACAAAGAAAACACCAAATAAGGTACCTGACGCAGCGGCAACACCACGTCGACTTGCTGGCGGCCGCTCTAGAACTAGτGGAT-3′; Nup2F, 5′-CATTGTTGGGGTCTCCGTGTTCTACACGTCCTTACTCCAGGTGAAGTGAGTGACGGGAAAGAAGAAAGGGGAAGGAAAACGTATAATGCAGACCTGCTGC-3′; Nup2R, 5′-CACTGTGAAATGCACGCACTGCTTCCACCACGCGTTCCTCCGCAGTTTCTGGCATTGCGCTTTCATTGCCCGCAGCGATCATACTACCCGATCCTGATCC-3′. Linear PCR products were purified and sterilized by ethanol precipitation and use for transfection.

### Immunofluorescence

For microscopy, cells were prepared for microscopy as previously described (Field et al., 2004). Briefly, cells were fixed with 3% paraformaldehyde (v/v) for 15 min at room temperature, washed and allowed to settle onto poly-L-lysine coated slides (VWR International) at room temperature. For permeabilization, cells were incubated with 0.2% Triton X-100 (v/v) in PBS for 10 min and washed three times with excess PBS. Slides were blocked in 20% FBS (Gibco) in PBS for at least 1 h. Cells were incubated with primary and secondary antibodies, successively with washes in excess PBS after antibodies incubations. Slides were mounted with mounting medium plus DAPI (Vectashield Labs). Primary antibodies were used at the following concentrations: anti-HA (1:1000; mouse Santa Cruz Biotechnology sc-7392 or rat Roche 11867423001); anti-Myc (1:400; monoclonal Millipore M4439), anti-TY1 (1:1000; monoclonal mouse Imprint SAB4800032); polyclonal rabbit anti-NUP-1 repeats (1:750; DuBois et al., 2012). Secondary antibodies were goat anti-mouse-IgG Alexa Fluor 488, goat anti-rabbit-IgG Alexa Fluor 568 and goat anti-rat-IgG Alexa Fluor 568 (Invitrogen, A11001, A11011, A11077, respectively) and were used at 1:1000. Confocal microscopy was carried out on a Zeiss microscope and images captured and deconvolved using Zen (Zeiss). For high-resolution microscopy, a Leica System microscope was used and images captured and deconvolved with LAS X software. Image analysis/preparation was made with the OMERO platform (Allan et al., 2012).

### Electron microscopy

Samples for electron microscopy were prepared using a modified protocol previously described (Gadelha et al., 2009). NUP-1 variant cells were induced at 1 μg/ml of Tet for 24 h. 2×10^7^ cells were harvested by centrifugation (800 ***g***, 10 min) and then resuspended in 0.5 ml of HMI-9 medium and fixed by the addition of isothermal glutaraldehyde to a final concentration of 2.5%. Cells were gently rocked for 10 min at room temperature (RT) culture then harvested at 2000 ***g*** for 2 min at RT and resuspended in 2.5% glutaraldehyde in PBS for another 30 min at RT. The samples were then post-fixed and processed at the University of Dundee Imaging Facility as previously reported (Maishman et al., 2016). Sections of 70 nm resin were used for imaging; images were taken in a JEOL 1200EX microscope using a SIS Megaview III camera running SIS software. Image analysis and preparation was undertaken with the OMERO platform (Allan et al., 2012).

### Immunogold localization

For immunogold labelling, 2×10^7^ cells of the following lines were used: SMB cells (control cells), cells expressing the N, C-terminal and N+C mutants (Tet-induced for 24 h). Cells were fixed with 4% formaldehyde with 0.1% glutaraldehyde in 0.1 M HEPES (pH 7.2) for 1 h at RT. After washing in HEPES with 20 mM glycine, pellets of cells embedded in 10% gelatin were immersed in 2.3 M sucrose for 24 h at 4°C and frozen by plunging into liquid nitrogen. Cryosections were cut using an EM UC6 ultramicrotome equipped with an EM FC6 cryochamber (Leica). Cryosections were picked up with a drop of 1.15 M sucrose and 1% methylcellulose. Sections were incubated in blocking solution (1% fish skin gelatine, Sigma-Aldrich) in HEPES with 20 mM glycine for 1 h at RT and incubated with anti-HA antibody, either rabbit (Sigma) or rat (Roche), diluted 1:40 in blocking solution for 15 min at RT. Sections were washed (six times, 2 min each) with blocking solution and incubated with protein A conjugated to 5 nm gold nanoparticles (UMC, Utrecht) diluted in blocking solution 1:50 for 45 min. Samples were washed in HEPES (six times, 2 min each) and dH_2_O, contrasted and embedded in 1.8% methylcellulose and 0.3% uranyl acetate. Samples were observed with a JEOL 1010 transmission electron microscope (TEM) operating at an accelerating voltage 80 kV and equipped with a MegaView III CCD camera (SIS). Refer to Fig. S4 for negative controls.

### Fluorescence *in situ* hybridization

Telomeres were detected using the PNA FISH kit (DAKO K5326) following the manufacturer's instructions. The probe for telomeres is coupled to Cy3. For combined immunofluorescence analysis and the Telomere PNA kit, cells were prepared for immunofluorescence analysis first following the protocol mentioned above. Briefly, after washing the secondary antibody, cells on the slides were fixed with 3.7% formaldehyde during 1 h at room temperature. Slides were then washed twice in TBS, immersed in pre-treatment solution and washed twice again. Slides were immersed in cold (−20°C) ethanol series (70%, 85% and 95%) and then dried. Telomere PNA probe (Cy3) was applied to the slides, moved into a pre-heated incubator at 80°C for 5 min and then placed in the dark at RT for 2–4 h. Slides were rinsed, washed and immersed again in the same cold ethanol series as previously. After drying the slides, DAPI were mounted with Vectashield mounting medium plus DAPI (Vectashield Labs). Confocal microscopy was carried out on a Zeiss microscope (Axiovert 200 M). Images were taken and deconvolved with Zen software (Zeiss). Image processing was performed with the OMERO platform (Allan et al., 2012).

### Proteomics

*T. brucei* bloodstream form cells expressing NUP-1 variants were cultured with 1.0 μg/ml of Tet for 24 h. SMB cells and uninduced cells were used as controls. Cells were washed with PBS containing Complete protease inhibitors (Roche), extracted with 1× NuPAGE sample buffer and sonicated. Lysates containing 10^7^ cells were fractionated on a NuPAGE Bis-Tris 4-12% gradient polyacrylamide gel (Thermo Scientific) under reducing conditions at 100 V for 10 min. The migration portion was contained in one slice that was subjected to tryptic digestion and reductive alkylation. Liquid chromatography mass spectrometry (LC-MS2) was performed in-house at the University of Dundee, UK. Samples were analysed on a Dionex UltiMate 3000 RSLCnano System coupled to a Q Exactive HF Hybrid Quadrupole-Orbitrap mass spectrometer (Thermo Scientific). Protein mass spectra were analysed using MaxQuant version 1.6.1.0 (Cox and Mann, 2008) searching the predicted *T. brucei brucei* TREU927 proteome (Aslett et al., 2010). Ratios were calculated from label-free quantification intensities (NUP-1 variant cell lines versus the uninduced control cells) using only peptides that could be uniquely mapped to a given protein. *P*-values were calculated applying *t-*test-based statistics using Perseus (Tyanova et al., 2016) and the −log*P* and FDR (0.01, s0=2) were calculated. Experiments were conducted in triplicate. Proteomics data have been deposited to the ProteomeXchange Consortium via the PRIDE partner repository (Vizcaíno et al., 2016) with the dataset identifier PXD019978. For the selection of differentially expressed proteins (those having abundance shifts after the overexpression of NUP-1 variants), we considered the following criteria: proteins containing at least 2 unique peptides, proteins with a statistical difference with respect to control cells ±0.3 and proteins with a statistical significance (log*P*) >1.5. For VSG221 quantification the data was re-analysed with the *T. brucei* Lister 427 as search database (Aslett et al., 2010). The repeat region of NUP-1 (absent from the ectopic constructs) was used to distinguish endogenous and ectopic NUP-1.

### TbNup98 silencing

RNAit (Redmond et al., 2003, https://dag.compbio.dundee.ac.uk/RNAit/) was used to design primers for TbNup98 RNAi. The sequences for primers were: Nup98F, 5′-AAGCTTGGGCCCCCCGGGATTCCTTTACGCCCACCTCG-3′ and Nup98R, 5′-AAGCTTTCTAGAGGATCCCTATCATCTGGGACCCACGC3′. PCR products were cloned into the pRPa^iSL^ plasmid (Alsford and Horn, 2008) into sites XmaI/BamHI and XbaI/ApaI. The construct generated was linearized with AscI and used for electroporation. Positive clones were assessed by qPCR.

### Flow cytometry analysis of DNA content

Cells expressing NUP-1 mutants were induced with 1 µg/ml Tet for 24 h. Cells were harvested, resuspended in 1% FBS in PBS and transferred to FACS tubes (Scientific Lab Supplies). Cells were fixed in ice cold 90% methanol for 30 min, washed twice in 1% FBS in PBS and finally resuspended in Staining Buffer (50 mg/ml propidium iodide, 50 mg/ml RNase A in 1% FBS in PBS). Samples were covered from light for 20 min. Samples were analysed for DNA content using a FACS Canto flow cytometer (Becton Dickinson) and DIVA acquisition software. Propidium iodide fluorescence was detected using 488 nm excitation and emission was detected at 585 nm±40 nm. Flow cytometry profiles for 10,000 propidium iodide-labelled cells post induction were obtained. Analysis of data and generation of histograms were performed in FlowJo version 10.6.2.

### RNA-seq analysis

Cells bearing the N+C construct were used for the transcriptomics assay. Cells were induced using 1 µg/ml of Tet (during 24 h) and SMB parental cells were used as control. 10^8^ cells were used for isolation of total RNA using a Macherey-Nagel NucleoSpin RNA kit (740955) as per manufacturer's instructions and eluted in high purity RNase-free water. The samples were sequenced in triplicates by Global Genomic Services (GBI). Sequencing resulted in paired-ended reads 2×100 bp, 12 million reads per sample. RNA-seq reads were mapped to the reference genome *T. brucei* TREU927, release 44, from TriTrypDB database. For VSG and procyclin genes, *T. brucei* 427 genome was used (Aslett et al., 2010). Mapping was done using STAR 2.6.0c aligner (Dobin et al., 2013); 70% of reads were mapped uniquely to the genome. Read counts per gene were found in the same STAR run, using TriTrypDB annotations in a GFF file. Data analysis was done in R environment. RNA-seq data are available in the NCBI BioSample database (http://www.ncbi.nlm.nih.gov/biosample/) under accession number PRJNA642306.

## Supplementary Material

Supplementary information
