# Supplementary material for: A hub-and-spoke nuclear lamina architecture in trypanosomes
Source: J Cell Sci. 2021 Jun 21;134(12):jcs251264. doi: 10.1242/jcs.251264 (PMC8255026; doi:10.1242/jcs.251264)
Supplement: Supplementary information [file joces-134-251264-s1.pdf]

Phyre²

Email	nepadillamejia@dundee.ac.uk
Description	NUP-1_repeats
Date	Fri Mar 20 14:07:09 GMT 2020
Unique Job ID	428f1c12be9033eb

Detailed template information

#	Template	Alignment Coverage	3D Model	Confidence	% i.d.	Template Information
1	c6h2xA_	Alignment	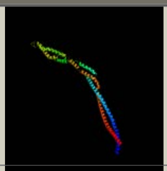	98.6	11	**PDB header:**dna binding protein<br>**Chain:** A: **PDB Molecule:**chromosome partition protein mukb,chromosome partition<br>**PDBTitle:** mukb coiled-coil elbow from e. coli
2	c1c1gA_	Alignment	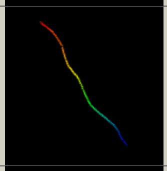	98.5	14	**PDB header:**contractile protein<br>**Chain:** A: **PDB Molecule:**tropomyosin;<br>**PDBTitle:** crystal structure of tropomyosin at 7 angstroms resolution in the2 spermine-induced crystal form
3	c5lm2B_	Alignment	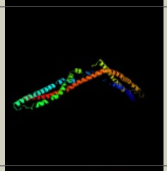	98.4	10	**PDB header:**hydrolase<br>**Chain:** B: **PDB Molecule:**tyrosine-protein phosphatase non-receptor type 23;<br>**PDBTitle:** crystal structure of hd-ptp phosphatase
4	c5xg2A_	Alignment	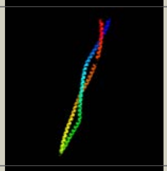	98.1	17	**PDB header:**dna binding protein<br>**Chain:** A: **PDB Molecule:**chromosome partition protein smc;<br>**PDBTitle:** crystal structure of a coiled-coil segment (residues 345-468 and 694-2 814) of pyrococcus yayanosii smc
5	c3wuqA_	Alignment	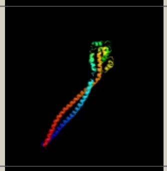	97.7	8	**PDB header:**motor protein<br>**Chain:** A: **PDB Molecule:**cytoplasmic dynein 1 heavy chain 1;<br>**PDBTitle:** structure of the entire stalk region of the dynein motor domain
6	c1ciiA_	Alignment	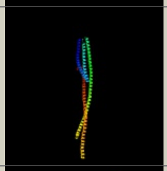	97.6	10	**PDB header:**transmembrane protein<br>**Chain:** A: **PDB Molecule:**colicin ia;<br>**PDBTitle:** colicin ia
7	c4cgkA_	Alignment	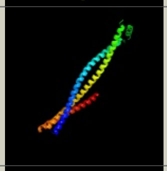	97.5	11	**PDB header:**cell cycle<br>**Chain:** A: **PDB Molecule:**secreted 45 kda protein;<br>**PDBTitle:** crystal structure of the essential protein pcsb from streptococcus2 pneumoniae
8	c5nnvD_	Alignment	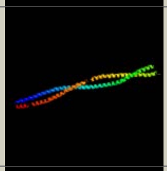	97.5	14	**PDB header:**cell cycle<br>**Chain:** D: **PDB Molecule:**chromosome partition protein smc,chromosome partition<br>**PDBTitle:** structure of a bacillus subtilis smc coiled coil middle fragment
9	c2oevA_	Alignment	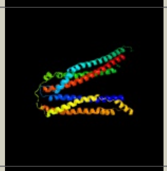	97.4	11	**PDB header:**protein transport<br>**Chain:** A: **PDB Molecule:**programmed cell death 6-interacting protein;<br>**PDBTitle:** crystal structure of alix/aip1
10	c5j1iA_	Alignment	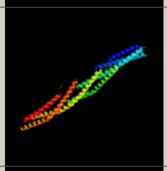	97.4	11	**PDB header:**structural protein<br>**Chain:** A: **PDB Molecule:**plectin;<br>**PDBTitle:** structure of the spectrin repeats 7, 8, and 9 of the plakin domain of2 plectin
11	c6gapB_	Alignment	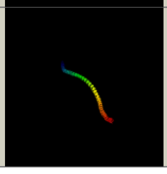	97.2	16	**PDB header:**viral protein<br>**Chain:** B: **PDB Molecule:**outer capsid protein sigma-1;<br>**PDBTitle:** crystal structure of the t3d reovirus sigma1 coiled coil tail and body

Fig. S1. Structural modelling of the NUP-1 repeat. The sequence corresponding to a single repeat was submitted to the Phyre2 server (http://www.sbg.bio.ic.ac.uk/phyre2) for modeling (Kelley et al., 2015). The intensive algorithm was selected.

Kelley, L. A., Mezulis, S., Yates, C. M., Wass, M. N. and Sternberg, M. J. E. (2015). The Phyre2 web portal for protein modeling, prediction and analysis. *Nat. Protoc.* **10**, 845-858. doi:10.1038/nprot.2015.053

Journal of Cell Science • Supplementary information

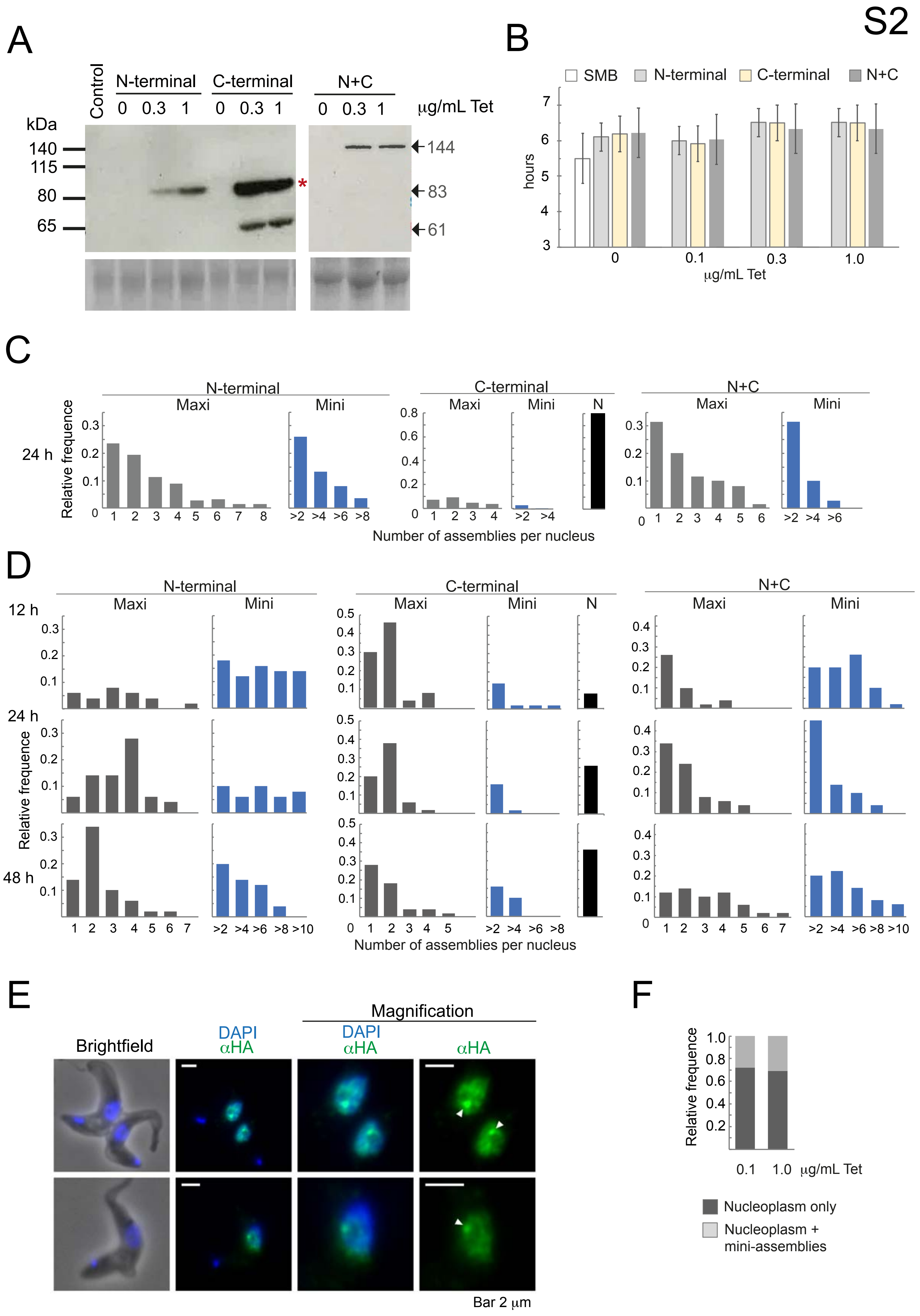

Journal of Cell Science • Supplementary information

Fig. S2. Characterization of NUP-1 overexpression mutants. (A) Detection of NUP-1 mutants by immunoblotting using an anti-HA antibody. Bands for N-terminal (83 kDa), Cterminal (63 kDa) and N+C (144 kDa) are shown. An extra band for C-terminal mutant is detected of ~80 kDa (red star). Ponceau staining of membranes is shown as loading control (B) Growth curves were followed across six days to assess the growth rate and the doubling times were calculated. SMB cells (parental line) and cells expressing the N-terminal, C-terminal and N+C mutants were used. Growth curves were assessed at 0, 0.1, 0.3 and 1.0 μg/ml of tetracycline for three different experiments. Cells were counted every 24 hours. The average doubling time and standard deviation were calculated. Error bar represents standard deviation. A gradual increment in the doubling time is observed at doses of 0.3 and 1.0 μg/ml of tetracycline. (C) Bar chart representing the number of maxi and mini assemblies per nucleus. Overexpression of NUP-1 mutants was induced during 24 h with 0.1 μg/ml Tet. Data for N-terminal, C-terminal and N+C fusion mutants is shown. The relative frequency of number of assemblies per nucleus is shown (n=50). For C-terminal mutant, the frequency of nucleoplasmic pattern (N) is also reported. (D) Effect of time in the formation of NUP-1 mutant assemblies. The relative frequency of number of assemblies per nucleus is shown (n=50). Overexpression of NUP-1 mutants was induced during 12 h, 24 h and 48 h with 1.0 μg/ml Tet. Data for N-terminal, C-terminal and N+C fusion mutants is shown. For C-terminal mutant, the frequency of nucleoplasmic pattern (N) is also reported. (E) Immunofluorescence analysis in cells bearing the C-terminal mutant after 24 hours of induction with 1.0 μg/ml of tetracycline. NUP-1 C-terminal mutants were probed with an anti-HA antibody (green). Miniassemblies (white arrowhead) occurring at the same time with the nucleoplasmic patter are shown. DAPI was used to visualize DNA. Scale bar 2 μm. (F) Bar chart representing the frequency of phenotypes with nucleoplasmic distribution during the expression of the C-terminal mutant. Cells with nucleoplasmic distribution from C and D (24 h) were taken. Two different phenotypes were found: i) one showing only nucleoplasmic distribution and ii) nucleoplasmic distribution plus mini-assemblies (≤5) as no maxi-assemblies were found to cooccur with the nucleoplasmic phenotype. Overexpression of C-terminal mutant was induced during 24 h with 0.1 and 1.0 μg/ml Tet. The relative frequency of phenotypes per nucleus is shown.

Journal of Cell Science • Supplementary information

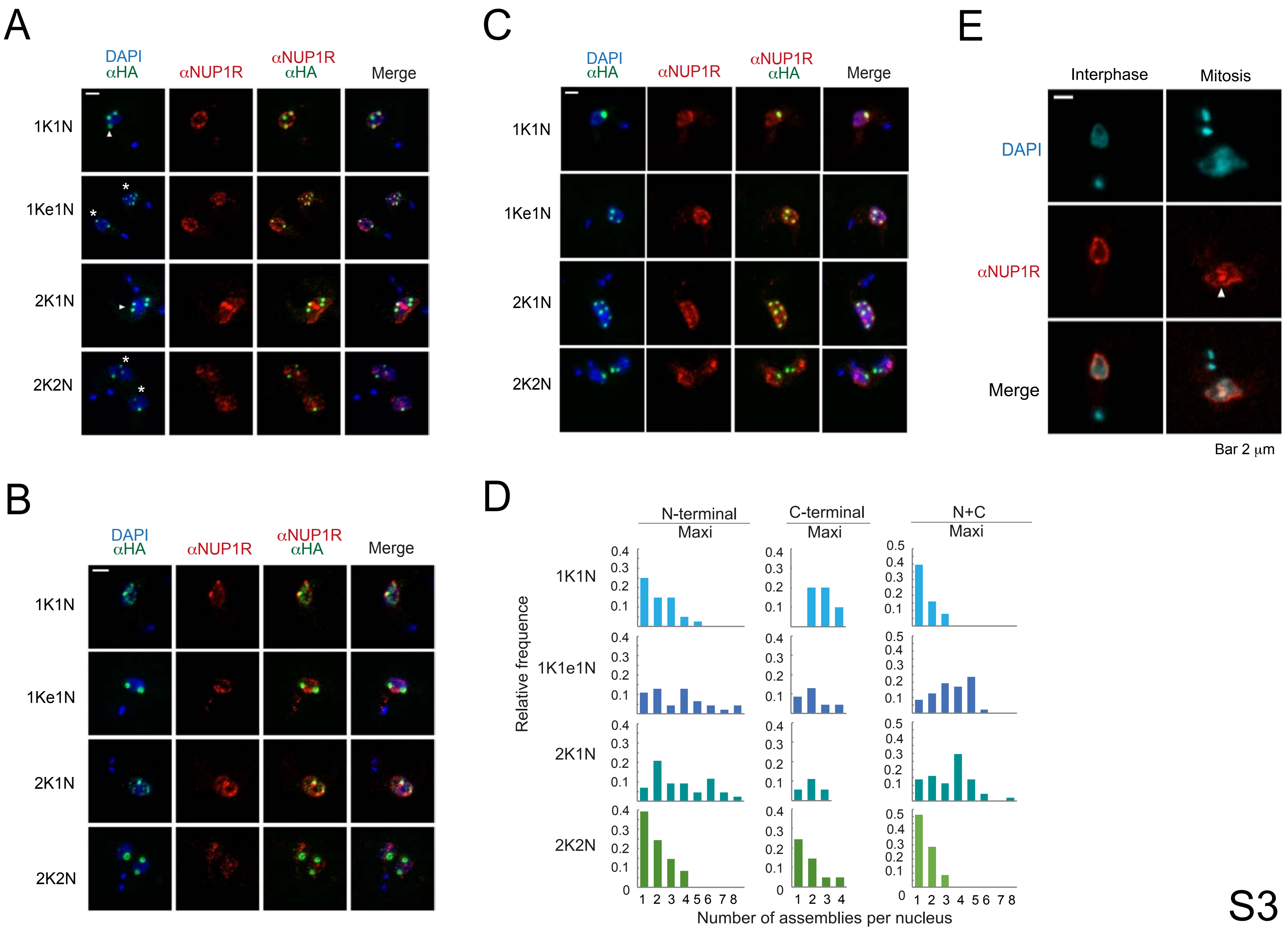

S3

Fig. S3. NUP-1 assemblies disrupt normal arrangement of endogenous lamina through the cell cycle. The associations between the assemblies and NUP-1 repeats were unaltered across the cell cycle. BSF cells containing the HA-tagged NUP-1 variants were fixed, stained and visualized by confocal immunofluorescence microscopy. Cells in different stages of cell cycle are shown. Cells were co-stained with anti-HA (green), anti- NUP-1 repeats serum (red) and DAPI (blue), as indicated. Central z-stacks are presen-ted. Scale bar 2 µm. (A) N-terminal mutant, (B) C-terminal mutant and (C) N+C mutant. In (A) maxi (white arrowheads) and mini-assemblies (white stars) are labelled in the first column (DAPI/anti-HA). (D) Effect of the cell cycle in the number of NUP-1 mutant maxi-assemblies. The relative frequency of number of assemblies per nucleus is shown (n=50). Overexpression of NUP-1 mutants was induced during 24 h with 0.1 µg/ml Tet. Data for N-terminal, C-terminal and N+C fusion mutants is shown. (E) NUP-1 α-helical repeats (NUP-1R) in SMB (parental cells) detected by Immunofluorescence (stained with anti-NUP-1 repeats serum, red). A cell in interphase and one in mitosis are shown. DAPI was used to visualize DNA. Scale bar 2 µm.

Journal of Cell Science • Supplementary information

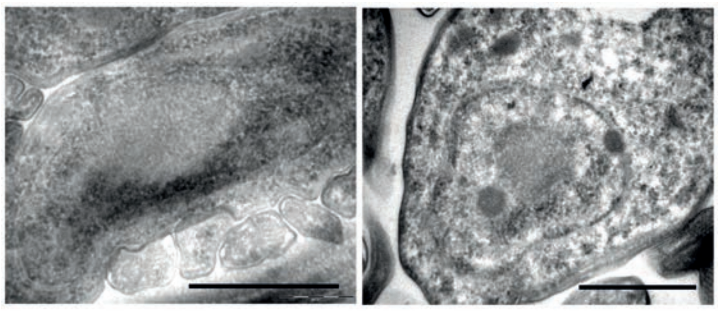

Fig. S4. Negative controls for immunogold localization. Left: SMB parental cells, Right: cells expressing N-terminal mutant, stained only with secondary antibodies. Scale bar 1 µm.

Journal of Cell Science • Supplementary information

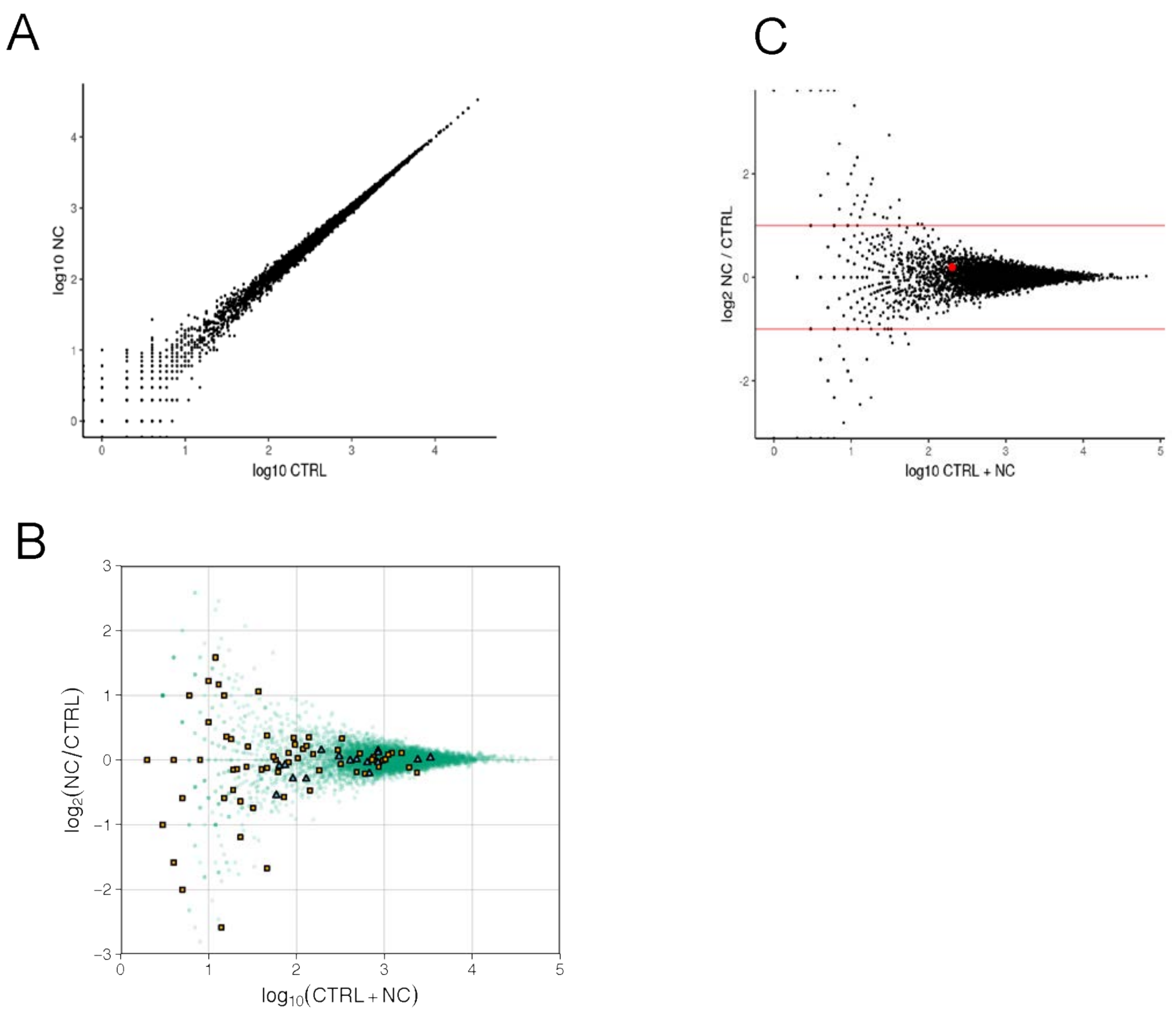

Fig. S5. Transcriptomics. A pilot RNAseq experiment was conducted using cells expressing the N+C fusion mutant and SMB cells as control. (A) Scatter plot of normalized RNA-seq read counts, showing no differentially expressed genes between the two conditions. Intensity-ratio plot of RNA-seq data. Plots based on raw counts per gene. There is no evidence for changes in the expression of the (B) intrinsic VSG-2 (VSG221 or Tb427.BES40.22), represented by a red dot nor (C) other VSG genes (orange squares) or procyclin genes (blue triangles). Green small points represent the rest of the transcripts across the genome. No evidence is found for any of the genes changing between conditions.

Journal of Cell Science • Supplementary information

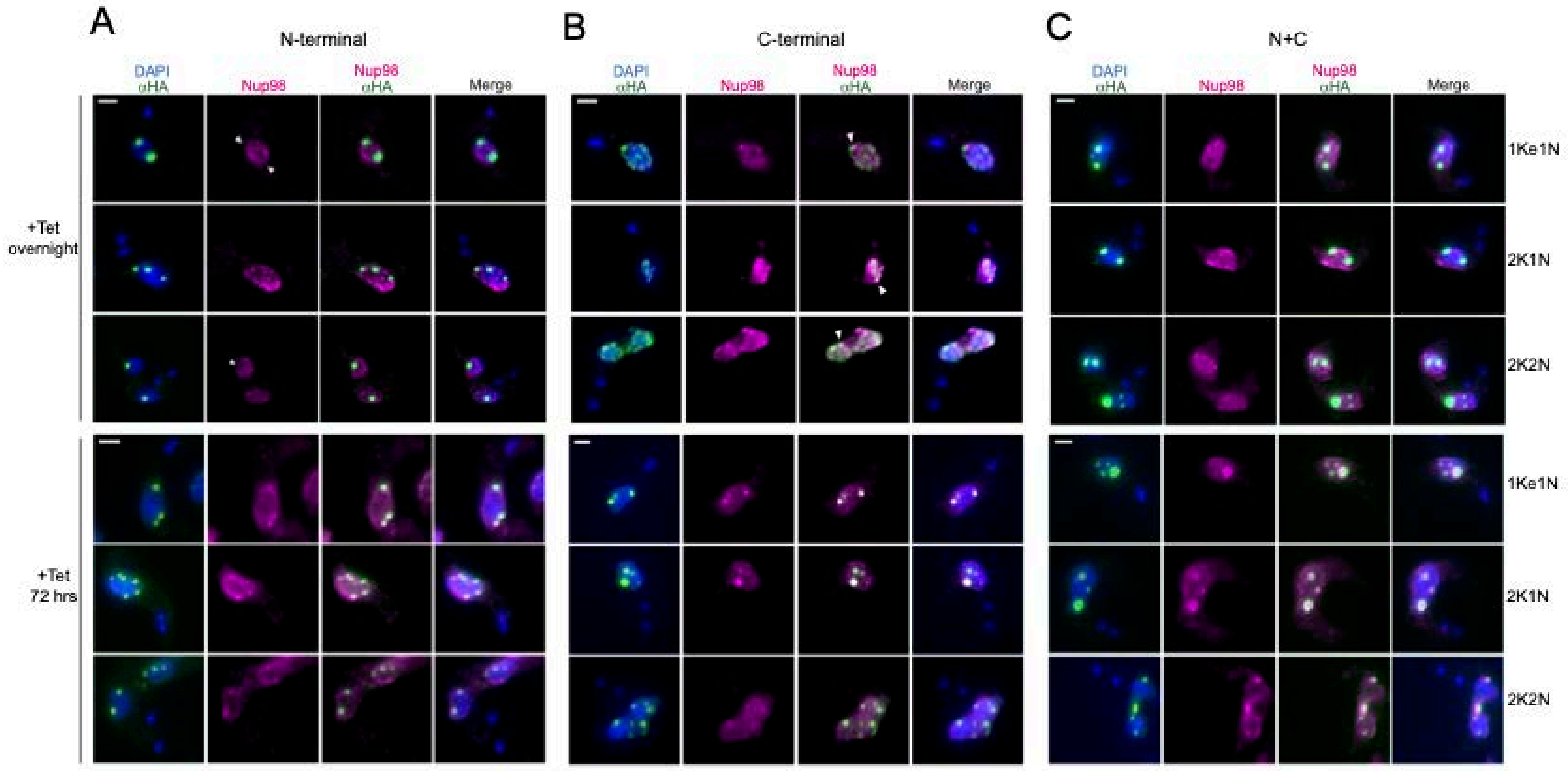

Fig. S6. TbNup98 interacts with both N-terminal and C-terminal domains of NUP-1 across the cell cycle. TbNup98::myc is visualised together with NUP-1 variants after two induction times with tetracycline: 16 (overnight) and 72 hours. Cells were fixed, stained as indicated and visualized by confocal immunofluorescence microscopy. Overexpressed NUP-1 domains are shown for the (A) N-terminal, (B) C-terminal and (C) N+C fusion variant. Cells are co-stained with anti-HA (green) and anti-myc antibodies (magenta). DAPI was used to visualize DNA (blue). After 16 hours induction, there are regions where TbNup98 is absent from the normal distribution (arrowheads) and do not interact with the NUP-1 assemblies. After 72 hours, NUP-1 mutants colocalize with TbNup98. Central z-stacks are shown. Scale bar 2 µm.

Journal of Cell Science • Supplementary information

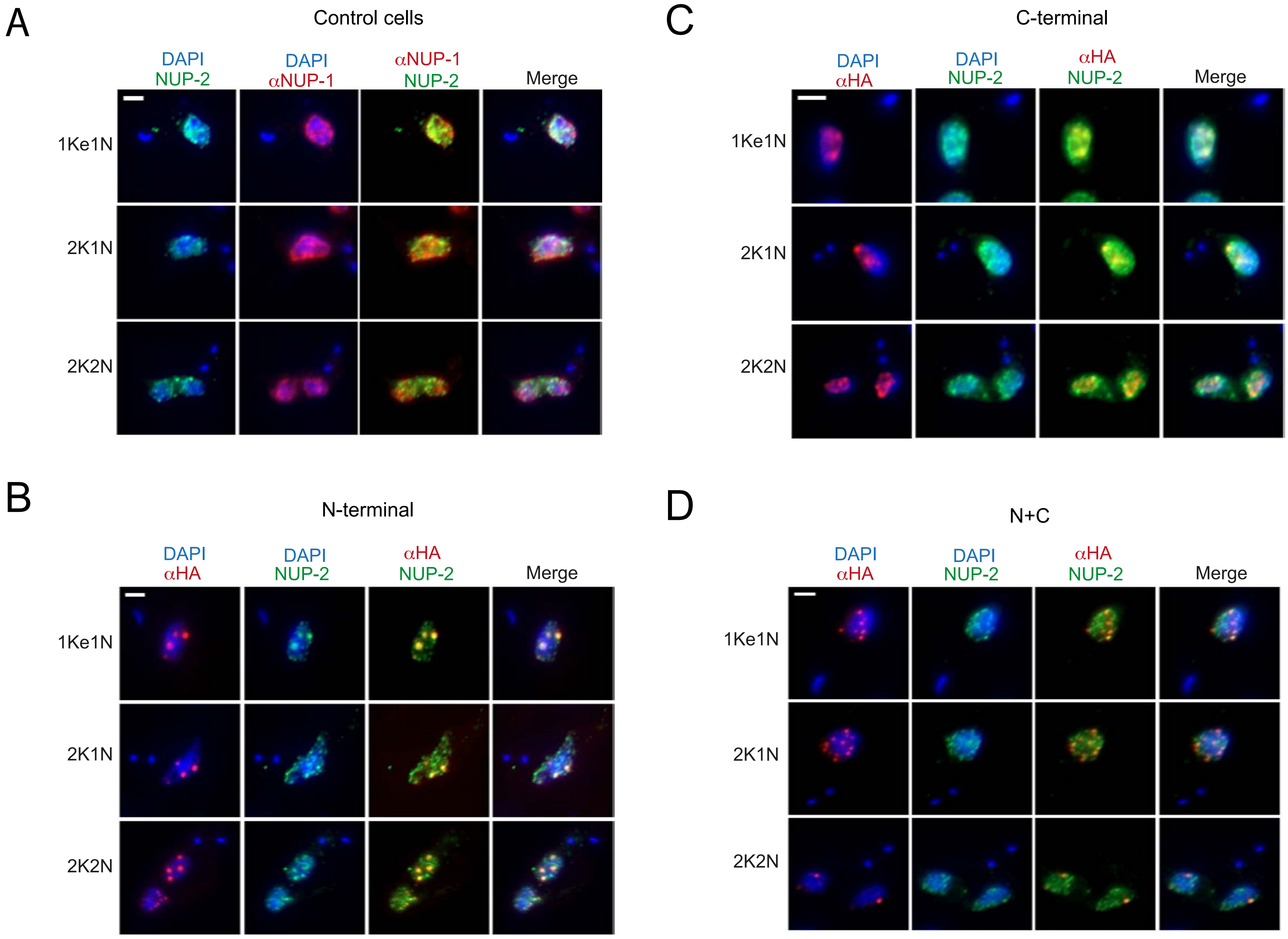

Fig. S7. NUP-2 interactions with NUP-1 across the cell cycle. NUP-2::TY1 is visualised together with endogenous NUP-1 and NUP-1 mutant variants. Cells were fixed, stained as indicated and visualised by confocal immunofluorescence microscopy. DAPI is used to visualise DNA (blue). (A) Parental SMB cells bearing NUP-2::TY1 were costained with anti-TY1 antibodies (green) and α-NUP-1 repeats serum (red). Cells expressing the NUP-1::HA variants were tet-induced and co-stained with anti-HA (red) andanti-TY1 (green) antibodies: (B) N-terminal, (C) C-terminal and (D) N+C fusion variant. Central z-stacks are shown. Scale bar 2 µm.

Journal of Cell Science • Supplementary information

```
ATGTTTTCTG CTGGGGACGC ACGGCGGTAC CCCGGTTTCT TCACACGAAC GTGGACTCCA     60
CCACCCGAAA ATATTGGGCA TGTGAGAAGC AACCGAAGCG CCAGCAGCAT CCAAGGAGGT    120
TTGACACATG AGACACCGCC GCTGCTCACA CCGCGGCTGG CGGCACCAAT TAATGTGCGA    180
GGATTGGCCG CAACGGATTC CATACCTCGT CTCAACAATC CTTTACCATC TCCTACGGGG    240
TTGCTGACTA ATTCTGCACT CGTCGGCATG TCACGTGAGG AACCCAGCAT AATGCCGCTT    300
CTCAGCTCCA AACAAACTGC TCCTATGGGA ATGCACCCCA CCATCTTCGC TGGGCGTCGG    360
GGCTTGGCGG ACATGTCAGA GGAGGAGCGC ATGGAGTACA CAAATCGCTT GGAGGGAGAT    420
ATGACTCATG TGCATAATAC CCTTAGCCGT GCGTATCAGT TGCGTGATGA CTACAAAAAT    480
GAGGCTGCAC GCCTCCATCG TGAGTTGCAG GACAAAAATC ATCGTTTTGA TTGCCTTTTG    540
CGCGAGCATA GTGCATGCAA CGACGTAATA TACCGCTGCA AGCGAGAAAA CGAAGAACTG    600
CGTCAAAAAC TTGACGAATC GGAAGGGGAG GTGCGACAGC TTAGGGATAA GCTAGTGTCC    660
GTAAATTCAC AAGGCAAATA TGTCCCATCT GGAGGTGAAC GACATGTTGG CCGTCAAGAG    720
ATAAGTGCGC TTGAAGAGAA GAACAAAAAA CTTGAGGAAG AACTTCTGGA GCTAACTAAA    780
GAGCTTGAAA GGGAACGTGA GTGTATTAGG CACCACGCTG TCGCTGCAGA AATGGGGAAA    840
AGTGAAAACA CGAGTCATGA AGAGGAATTG GCGCAATCAA GGTACCTGTT ACAGGTGACA    900
CGTACAGAGA TAACGGATCT ACAGCAGCTT CTTCGAAAAG AACGTGAAGA CTACGAAGAA    960
AGCCTTAGGG AAGCAATACA AGCGCGAAAC AACCTTCACC AACAAAACAC CGCACTGCAG   1020
GAGCAAAAGG AACAATTGCA GGAAATGTGT GACGAACAGC ACAGGACGAT TGAGGATCTT   1080
ACTTCACAAC TACTACAGCG CAAAACTGAG CAAGCAGTAC AACGTGGGGC ACCTGACACA   1140
CAAATGGAGA CAACAGACGA GAACAAAACA GACACAAATA CAAACAACGA TGATGAAGTG   1200
TACAGAATGC TGGAACTACA ACAGCACACA CTTCAACAGC AGTTCTTCTT GCTCCGTAGG   1260
GAAGGAGAAG CGAAGGACAT CCTGCTCCAA AAAGCGAGCG AAGAAATATT TAACCTCCAA   1320
AATCTTCAGC AGCAACTAGA GGCCGCCCTT CAGAAATCGA GAGAACACGC TGCAGAACTA   1380
ACGAAAAGCC TTTCCCACAC ACAAAACCAA CTGCAGACTG CTCAAGAAAG GATCACCGAA   1440
GATAGCTATG TGATCAACAA CTTTCATCAT CAACTCAGAG AAAAAATACA AATATCAGGC   1500
TCCATAAGCG GTGAAAAGAA CATTCCACAA GGCGGGAATA AAGAGGAATC AATAGAGTTA   1560
GTGACAAGAG AGACACAGAT GCCATCAAGG TCAGGAAATG ATTCGCAATA TATTACTGCA   1620
AATGTGCAAC ATGAGAAACT AAACCAACCA CAAAAAGCCG ATAGCGGTCA CAATGCCACA   1680
GGGAATAACA AGGAATTGTC GTCAGCTCAA AATGACGAGT ACGAACAAGC CATAATCAAG   1740
CACAAGATGA CAGAAGAAGG TTTAACAGAA GTCATAGAAG CACTGAAAAC GGAACTGCAG   1800
CACACACAGA AGTGCCTTCG CGAGGCAGGG GAAGAGAACG TGCAACTAAC GAATAAGCTG   1860
AATGCGGCTG GAGCTCGGGG CCGCTCAACA AGTACAACAA GAAGTGGAAG TTTAACACCA   1920
AATGATACAG AAGGATCACT GAGAACATAT AATGCCGGAT TGAAAACACA GTTATCCTCC   1980
GCCTTGGCAG CCCTAACACA GCTAGCAGAA CAGCACGATG CTACACTAGC AAGAGCAACT   2040
GAAATGGAGG AACGTGTTTC TACACTTGAG GAGGAACTCC GTACAGCGCA CTCAACCACG   2100
//
GATCTTGGTA CACAATTGGC ATCTGCTTTA GTAGCACTTG AGCGACTTGC TGAGGAACGG   9360
GAAGCTGCTT TAGAAAAGGC AACTGAGATG GAGGAACGTG TTTCTACACT TGAGGAGGAA   9420
CTCCGTACAG CAAAGGAGAA GCTGGAGAGG AGTGTTGAGG AAATATCTTT TTTAAAAGAT   9480
GAAGTTTTGG TTAGTAATCG TTTGCTTGTG GATAGTGTTT CTTCTTTGAA TGGTAAAGTG   9540
GGGGATAGTG ATGGTGCTGT TGGTGCAGAT GTTGAGAGGT TGTCTCGGGT TGTGGATGAA   9600
CTTCATGCTC AAGTTTCCGC TACGAAGCGT GGTTTTGAAG AATTTTATGA CCGTAGGAGT   9660
GAGGGTTGCG TGACGGAACT TATTGTCGCA AGAAGATCCG TTGATCGCTC TAATGATGCG   9720
AGGAGGAGGT TGGAGGAGCG CAATGTTCGG CTAGAGCAAG ATTTGGAAAG AAAATGTCTG   9780
GAAGTAGTTA AATTGCAGAA GGAGTGCCAG CGGTTGGAAC AATTTGTTCG GGCAAAGGAC   9840
GTTCGTGGCG CGCACAGTGT ATTAGGTGTG GATGGTTCCG TTGATGTGAG TTCTGTGGGG   9900
GCGGAACCGG TGGACTTAGA GGCTGTAGAT CTCGCCCAGT TTCTACAAAT ATCAAGCCTT   9960
CACGCAGATC TAATGCTCTG CCGAAAAACT TGCCGTCAGT TGGAATCCAA TCAAGAGGAA  10020
CTTCTCTTGT CATTGGAACA GAATTCGTCG CAGTCAAATG CGTATCTGGA AGATTTAGAT  10080
GAAATTCGTC AACAGTTGGT GGAAATGCGT CAGCAACGTG AAGAACTCAT AGCTGAGCGT  10140
CGTACTCTCA CCGAGAGGGT CGATGAACTT GGTCGTGAGC GAGGTGAGGA AGTTAGTCGG  10200
TTGAAACAGC AGAACAACTC GCTCTCCGCA CAGTTGCAAG CGAGCCGCAA TAAACTCTCC  10260
GCACTGGAGG CATCGAAACG TGAGGGTGAA CTTGCGGCCA GGCAGCAAGC TGAGGAACTG  10320
GCGAAAGCAT TCAGTCTGAT GGAGGCTCAG GTGCAAACAC TCCGCGAGGA GGTTGCATCG  10380
ACGAGTGGTT CCCCAAAACG GCAAAGCGGT TCCTCTCGTC AAAAGGCCGT TGTGGAGGGG  10440
GATGAAGCGC GTATTCGCAT GTCGCAGGCG CGCGTTACGT TCCTTGAAAA AGCTCTACAA  10500
CGAAAGGATG AGGAGGTTCA ACGGCTGCAG GATGAGCTTG TACAGAAGGA CGAACAACTT  10560
GACCAGTATG AACAAGATGC GGCCAAGGCG GCACAAGATG CGGAGAATGC ATCAAGGAAG  10620
ACCTTACAAC TTGAAAGCGC AGTTCAGAAG TTGCAGGGCG ATAAGAAAGG TCTGGAGGAC  10680
GAGCTTCGAT ATGCCAAGAC AAGGGTTGTA ACCTATGGTG GTCGTGTGTC ATCAGAAGTG  10740
GCACAACACA GCAGCCCACC GGAACAGCAA ATTCGGGGGT CACCTGTGCT AGGTGCAGGA  10800
AGAACCACCA GAGAGAGGGT GAGCTTGTCC GTTGAGTCAT CACATCATTC CAGAATCACT  10860
GAACAAACAC AGCGACAGGT ACGGCAAGTC ATGGACATAC GTAGCACAAG GAAAAGGTCT  10920
CGTTCAGCCA ATGCGGTCTC GTGA                                         10944
```

Fig. S8. NUP-1 sequences used to create NUP-1 variants. NUP-1 (Tb927.2.4230) gene sequence is shown above with a gap in the central region of the gene. The N-terminal region is shown in blue, the initial start codon is underlined. The C-terminal region is shown in green with the endogenous NLS highlighted in yellow. The stop codon is shown in red. The N-terminal variant was built cloning the N-terminal sequence shown here followed by the native NLS. The C-terminal variant was built using the sequence in green, avoiding the stop codon. For the N+C variant a fusion of both sequences was made. In all mutants, the HA tag sequence was added at the end.

Journal of Cell Science • Supplementary information

Table S1. Excel sheet. Data from label-free quantitative mass spectrometry of whole lysates of cells expressing the NUP-1 constructs.

Click here to download Table S1

Table S2. List of differentially upregulated proteins in cells expressing NUP-1 constructs

	Exclusive N-terminal
Gene ID	Product Description
Tb927.10.5250	zinc finger protein family member, putative
Tb927.10.11300	paraflagellar rod component, putative
Tb927.10.11760	pumilio/PUF RNA binding protein 6
Tb927.10.12820	hypothetical protein, conserved
Tb927.10.2610	Domain of unknown function (DUF1935), putative
Tb927.10.3230	CMGC/MAPK protein kinase, putative
Tb927.10.3810	Nucleoporin NUP65
Tb927.11.13870	uncharacterized protein, PH0010 family/AmmeMemoRadiSam system protein A, putative
Tb927.11.15030	small GTPase
Tb927.11.3360	Component of motile flagella 22
Tb927.11.3500	Dpy-30 motif containing protein, putative
Tb927.11.5650	replication factor C, subunit 1, putative
Tb927.2.3580	transcription elongation factor s-II, putative
Tb927.2.4330	paraflagellar rod protein 5, putative
Tb927.3.1040	cAMP Response Protein 4
Tb927.3.1800	hypothetical protein, conserved
Tb927.3.4190	endosomal integral membrane protein, putative
Tb927.3.5020	Flagellar Member 6
Tb927.6.1080	hydroxyacylglutathione hydrolase, putative
Tb927.6.3100	Intraflagellar transport complex B protein 46 C terminal, putative
Tb927.7.3630	TPR-repeat-containing chaperone protein DNAJ, putative
Tb927.7.4750	hypothetical protein, conserved
Tb927.9.11540	hypothetical protein, conserved
Tb927.9.2390	hypothetical protein, conserved
	Exclusive C-terminal
Gene ID	Product description
Tb927.1.1540	Tubulin/FtsZ family, putative
Tb927.10.10140	paraflagellar rod component, putative
Tb927.10.11800	Axonemal inner arm dynein light chain, putative
Tb927.10.12360	hypothetical protein, conserved
Tb927.10.15850	Peroxisome biogenesis factor 12
Tb927.10.350	protein kinase PK4, putative
Tb927.10.5630	hypothetical protein, conserved
Tb927.10.5880	Proteophosphoglycan, putative
Tb927.10.6670	dynein light chain, putative
Tb927.10.7880	Sperm tail/Sperm tail C-terminal domain containing protein, putative
Tb927.10.8650	ran binding protein, putative
Tb927.10.8930	paraflagellar rod component, putative
Tb927.11.10540	hypothetical protein, conserved
Tb927.11.15480	heat shock protein Hsp20, putative
Tb927.11.15910	iron superoxide dismutase
Tb927.11.16090	Outer dynein arm docking complex protein 2, putative
Tb927.11.4450	ALBA-Domain Protein
Tb927.11.4920	hypothetical protein, conserved
Tb927.11.510	RNA-binding protein, UBP2, UBP1
Tb927.11.6280	pyruvate phosphate dikinase
Tb927.11.6370	leucine-rich repeat protein (LRRP), putative
Tb927.11.8440	haloacid dehalogenase-like hydrolase, putative
Tb927.11.9470	ADP-ribosylation factor GTPase activating protein, putative
Tb927.2.1890	E2-like ubiquitin-conjugation enzyme
Tb927.2.5810	Holliday-junction resolvase-like of SPT6/SH2 domain containing protein, putative
Tb927.3.1010	hypothetical protein, conserved
Tb927.3.3690	flagellar radial spoke protein-like, putative
Tb927.3.5010	hypothetical protein, conserved
Tb927.4.750	50S ribosomal protein L7Ae, putative
Tb927.5.2620	hypothetical protein, conserved
Tb927.5.4150	hypothetical protein, conserved
Tb927.6.1730	hypothetical protein, conserved
Tb927.6.3160	splicing factor 3a, putative
Tb927.6.3720	hypothetical protein, conserved
Tb927.7.1120	trypanothione/tryparedoxin dependent peroxidase 1, 3
Tb927.7.1310	hypothetical protein, conserved
Tb927.8.1340	60S ribosomal protein L7a, putative
Tb927.9.3770	hypothetical protein, conserved
Tb927.9.6560	NAK family pseudokinase, putative
Tb927.9.9940	parkin coregulated gene protein
	Exclusive N+C
Gene ID	Product Description
Tb927.11.3510	hypothetical protein, conserved
Tb927.11.6510	40S ribosomal protein S21, putative
Tb927.3.4920	LETM1 and EF-hand domain-containing protein 1, putative
Tb927.5.1250	GAF domain/TIP41-like family, putative
Tb927.5.2900	histone deacetylase 4
Tb927.9.10790	hypothetical protein
Tb927.9.13320	hypothetical protein, conserved
Tb927.9.1600	hypothetical protein, conserved

Click here to download Table S2

Journal of Cell Science • Supplementary information

Table S3. List of differentially downregulated proteins in cells expressing NUP-1 constructs.

	Exclusive N-terminal
Gene ID	Product description
Tb11.02.5380	exosome complex exonuclease RRP44p homologue
Tb11.v5.0480	DNA-directed RNA polymerase, alpha subunit, putative
Tb927.10.12630	hypothetical protein, conserved
Tb927.10.1320	hypothetical protein, conserved
Tb927.10.14840	Mitochondrial ADP/ATP carrier protein 5a
Tb927.10.3150	N-acetyltransferase, putative
Tb927.10.4000	methylglutaconyl-CoA hydratase, mitochondrial precursor, putative
Tb927.10.4740	nucleolar RNA-binding protein, putative
Tb927.10.5480	60S ribosomal protein L24, putative
Tb927.10.6970	serine peptidase, Clan SC, Family S9B
Tb927.10.730	ATP synthase, putative
Tb927.10.8830	Flagellum attachment zone protein 5
Tb927.10.9430	phosphoribosylpyrophosphate synthetase, putative
Tb927.11.13010	hypothetical protein
Tb927.11.14430	proteasome regulatory non-ATP-ase subunit
Tb927.11.14780	phosphomannose isomerase
Tb927.11.2260	Eukaryotic translation initiation factor 4E-1
Tb927.11.2400	Flabarin-like protein
Tb927.11.2730	UDP-galactose 4-epimerase
Tb927.11.800	prefoldin subunit, putative
Tb927.2.6150	adenosine transporter 2
Tb927.3.2230	succinyl-CoA synthetase alpha subunit, putative
Tb927.5.1160	Degradation arginine-rich protein for mis-folding, putative
Tb927.5.590	protein phosphatase 1, regulatory subunit, putative
Tb927.7.1670	Eukaryotic translation initiation factor 4E type 6
Tb927.7.2790	Component of motile flagella 10
Tb927.7.3080	Kinetochore interacting protein 4
Tb927.7.4440	NAD dependent epimerase/dehydratase family, putative
Tb927.7.5300	C2 domain/Ankyrin repeats (3 copies), putative
Tb927.8.730	nucleolar RNA-binding protein, putative
Tb927.9.10690	Protein of unknown function (DUF2009), putative
Tb927.9.11410;Tb927.9.11380	60S ribosomal protein L23, putative
Tb927.9.3990;Tb927.9.3920	ribosomal protein S7, putative
Tb927.9.8720	fructose-1,6-bisphosphatase

	Exclusive C-terminal
Gene ID	Product description
Tb927.1.1420	conserved protein, unknown function
Tb927.10.12510	P-type H+-ATPase, putative
Tb927.11.10030	60S ribosomal protein L29, putative
Tb927.11.1090	calpain-like protein, putative
Tb927.11.11830;Tb927.11.11820	40S ribosomal protein S17, putative
Tb927.11.11980	cytoskeleton-associated protein 15
Tb927.11.13970	Pab1p-dependent poly(A) ribonuclease subunit, putative
Tb927.11.14560	Cleavage and polyadenylation specificity factor CPSF160, putative
Tb927.11.14690	Microtubule-binding protein MIP-T3, putative
Tb927.11.16760	T-complex protein 1, alpha subunit, putative
Tb927.11.2250	conserved protein, unknown function
Tb927.11.230	cleavage and polyadenylation specificity factor subunit 2
Tb927.11.4820;Tb927.10.14580	60S ribosomal protein L17, putative
Tb927.11.740	eukaryotic translation initiation factor 5A
Tb927.2.1330	retrotransposon hot spot protein 6 (RHS6), degenerate
Tb927.2.2230	hypothetical protein, conserved
Tb927.3.3330	heat shock protein 20, putative
Tb927.3.4260	Acetyl-CoA hydrolase
Tb927.3.5580	tryptophanyl-tRNA synthetase
Tb927.4.2170	hypothetical protein, conserved
Tb927.4.2530	hypothetical protein, conserved
Tb927.4.4360;Tb927.8.8020	monoglyceride lipase, putative
Tb927.5.1260	Sulfate transporter N-terminal domain with GLY motif/Sulfate transporter family, putative
Tb927.6.1140	dolichyl-P-Man:GDP-Man5GlcNAc2-PP-dolichyl alpha-1,2-mannosyltransferase, putative
Tb927.6.1650;Tb927.6.1640	single strand-specific nuclease, putative
Tb927.6.2170	co-chaperone GrpE, putative
Tb927.6.3840	reticulon domain protein
Tb927.6.4090	chaperonin HSP60, mitochondrial precursor, putative
Tb927.7.240;Tb927.7.230	40S ribosomal protein S33, putative
Tb927.7.4220	WD domain, G-beta repeat/Dip2/Utp12 Family, putative
Tb927.7.7460	hypothetical protein, conserved
Tb927.8.4640	Component of motile flagella 19
Tb927.8.6070	Trypanosome basal body component protein
Tb927.8.8230;Tb927.8.8220	amino acid transporter, putative
Tb11.v5.0621	hypothetical protein, conserved
Tb927.9.1780	sec1 family transport protein, putative
Tb927.9.5410	hypothetical protein, conserved
Tb927.9.9670	proteasome alpha 1 subunit, putative

	Exclusive N+C
Gene ID	Product description
Tb927.10.5520	AmmeMemoRadiSam system protein B, putative
Tb927.11.5590	Anaphase-promoting complex-associated protein AP1
Tb927.4.2380	sarcoplasmic reticulum glycoprotein, putative
Tb927.11.9810	NUDIX hydrolase 3, putative
Tb927.10.260	BolA-like protein, putative
Tb927.11.16610	zinc-finger of a C2HC-type, putative

Click here to download Table S3

Journal of Cell Science • Supplementary information
